# Host Cell Entry of Respiratory Syncytial Virus Involves Macropinocytosis Followed by Proteolytic Activation of the F Protein

**DOI:** 10.1371/journal.ppat.1003309

**Published:** 2013-04-11

**Authors:** Magdalena Anna Krzyzaniak, Michael Thomas Zumstein, Juan Atilio Gerez, Paola Picotti, Ari Helenius

**Affiliations:** Institute of Biochemistry, ETH Zurich, Zurich, Switzerland; Johns Hopkins University, United States of America

## Abstract

Respiratory Syncytial Virus (RSV) is a highly pathogenic member of the Paramyxoviridae that causes severe respiratory tract infections. Reports in the literature have indicated that to infect cells the incoming viruses either fuse their envelope directly with the plasma membrane or exploit clathrin-mediated endocytosis. To study the entry process in human tissue culture cells (HeLa, A549), we used fluorescence microscopy and developed quantitative, FACS-based assays to follow virus binding to cells, endocytosis, intracellular trafficking, membrane fusion, and infection. A variety of perturbants were employed to characterize the cellular processes involved. We found that immediately after binding to cells RSV activated a signaling cascade involving the EGF receptor, Cdc42, PAK1, and downstream effectors. This led to a series of dramatic actin rearrangements; the cells rounded up, plasma membrane blebs were formed, and there was a significant increase in fluid uptake. If these effects were inhibited using compounds targeting Na^+^/H^+^ exchangers, myosin II, PAK1, and other factors, no infection was observed. The RSV was rapidly and efficiently internalized by an actin-dependent process that had all hallmarks of macropinocytosis. Rather than fusing with the plasma membrane, the viruses thus entered Rab5-positive, fluid-filled macropinosomes, and fused with the membranes of these on the average 50 min after internalization. Rab5 was required for infection. To find an explanation for the endocytosis requirement, which is unusual among paramyxoviruses, we analyzed the fusion protein, F, and could show that, although already cleaved by a furin family protease once, it underwent a second, critical proteolytic cleavage after internalization. This cleavage by a furin-like protease removed a small peptide from the F1 subunits, and made the virus infectious.

## Author Summary

Respiratory Syncytial Virus (RSV) is a highly pathogenic paramyxovirus. We developed assays for RSV endocytosis, intracellular trafficking, membrane fusion, and infection. The results showed that RSV was rapidly and efficiently internalized, and that acid-independent membrane fusion occurred intracellularly after endocytosis. Cell biological studies demonstrated that endocytosis was macropinocytic, and that it was required for infection. The process involved activation of the EGF receptor and its downstream effectors including Cdc42, Pak1, and myosin II. RSV induced transient actin rearrangements accompanied by plasma membrane blebbing, elevated fluid uptake, and internalization of intact RSV particles into large macropinosomes. Expression of a dominant negative Rab5 mutant but not Rab7 decreased infection indicating that RSV penetration is intracellular, and takes place in Rab5 positive macropinosomes before fusion with endolysosomal compartments. The reason why RSV, unlike most paramyxoviruses, depended on endocytic entry was found to be the need for activation of the F protein by a second proteolytic cleavage. It occurred after endocytosis, and involved most likely a furin-like, vacuolar enzyme.

## Introduction

Human respiratory syncytial virus (RSV) belongs to the Paramyxoviridae, a family of enveloped viruses with a negative-stranded RNA genome. It is a ubiquitous human pathogen that causes severe respiratory tract infections affecting mainly children and the elderly worldwide. Despite ongoing efforts, there are no available vaccines or treatments except passive immunoprophylaxis [1]. A better understanding of virus/host cell interactions is critical for the development of new therapeutic strategies.

RSV particles produced in tissue culture are heterogeneous in size and shape. Some are rounded with a diameter of 100–300 nm, others filamentous with a length up to 10 µm [2]. The nucleocapsid is helical and contains in addition to the RNA the nucleoprotein N, the viral polymerase L, its cofactor-phosphoprotein P, and the transcription processivity factor M2-1. The matrix protein M is believed to form a layer on the inside of the viral envelope [3]. The lipid envelope is derived from the plasma membrane (PM) of the infected host cell, and contains three viral glycoproteins; the major attachment protein G, the fusion protein F, and a small hydrophobic protein SH.

Cell attachment of RSV is mediated by G and F, which bind to cellular glycosaminoglycans [4]. That G and SH are not essential for replication in cell culture [5], indicates that the F protein can support both attachment and fusion. *In vivo*, RSV targets airway epithelial cells, and in the human mucociliary epithelium it infects ciliated cells from the apical surface [6].

Previous studies on RSV entry employing a lipid-dequenching assay suggested that RSV, as most other paramyxoviruses, fuses its membrane directly with the PM of target cells [7]. That RSV entry is pH-independent is consistent with this view [8]. On the other hand, Kolokoltsov and coworkers concluded, that RSV uses clathrin-mediated endocytosis (CME) to infect HeLa cells because a targeted siRNA screen revealed clathrin light chain, Eps-15, and AP-2 as important cellular factors in RSV infection [9]. In a recent publication, San-Juan-Vergara et al. [10] argued that in primary NHEB cells RSV entry is a two-step process; RSV docks to cholesterol-rich PM domains facilitating hemifusion between the viral envelope and the PM followed by endocytosis and complete fusion in endosomes.

To determine the pathway of RSV entry into HeLa and A549 cells, we developed quantitative fluorescence-activated cell sorting (FACS) assays and complemented them with confocal microscopy to monitor cell binding of RSV, endocytosis, fusion, and infection. We tested the effects of inhibitors and other perturbants. Our results indicated that RSV infected the cells by an endocytosis-mediated mechanism that fulfilled the criteria of macropinocytosis. After uptake into macropinosomes, a second proteolytic cleavage in F served as a ‘cue’ for penetration by membrane fusion.

## Results

### Purified RSV is efficient in cell binding and infection

**Figure 1:**
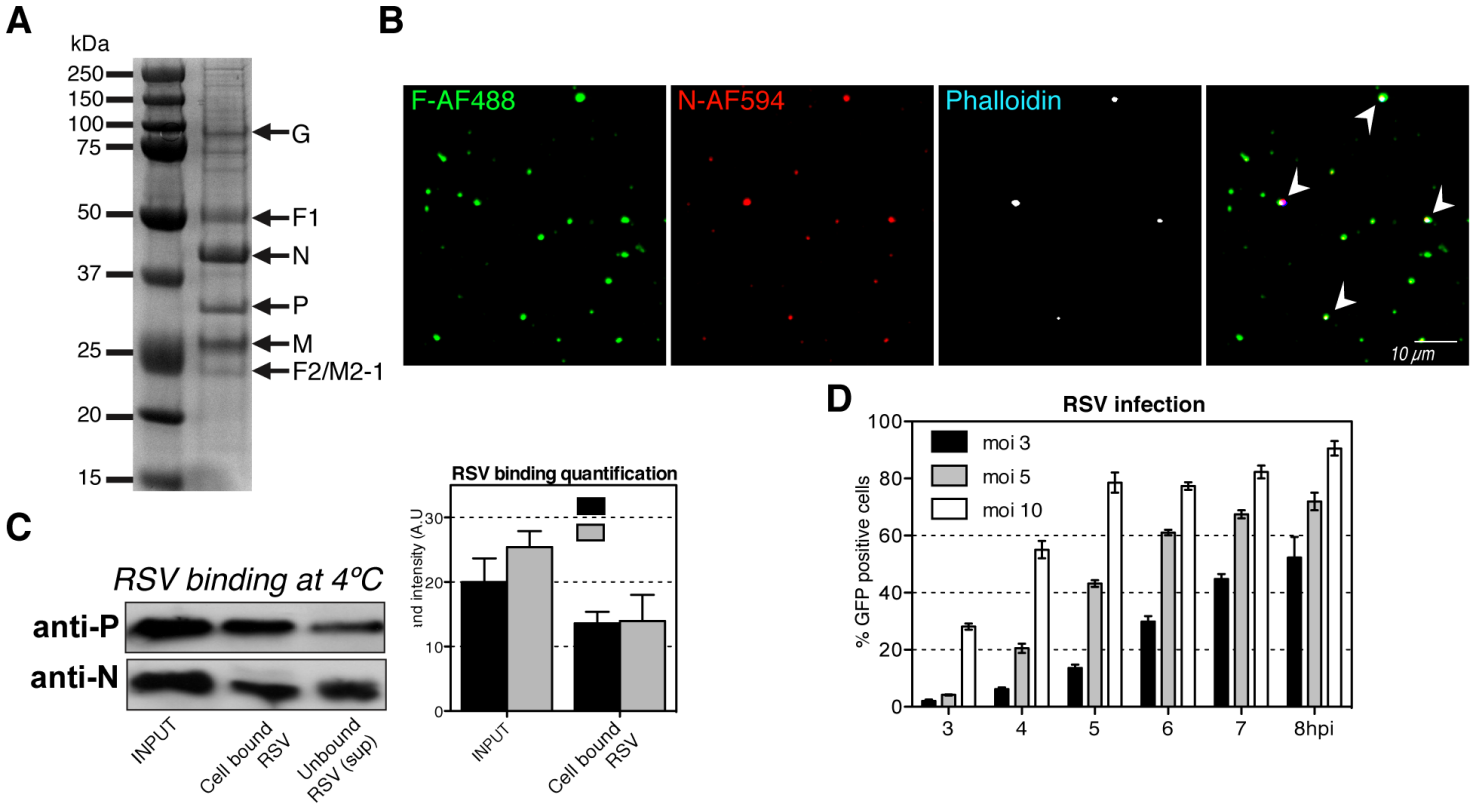
Purified RSV is efficient in cell binding and infection. (A). Gradient purifier RSV (∼10^5^ particles/50 µl) was resolved on the SDS-PAGE, followed by the blue silver gel staining. (B). After binding to a glass slide, purified RSV was stained with anti-F-AF488 (green), anti-N-AF594 (red), and phalloidin-AF647 (pseudocolored white). The particles (n = 457) were imaged with a confocal microscope and analyzed for colocalization by Imaris. Arrowheads show particles with all three stains. (C). Equal volumes of the virus input (moi 10), the cell bound virus lysates, and the unbound virus (sup) were resolved by a SDS-PAGE. Western blots were developed with anti-P or anti-N RSV specific antibody. (left) Representative western blots. (right) Densitometry quantification of the P- and N- protein bands intensities for the virus input and cell bound virus samples. (D). HeLa cells were infected with RSV moi (3–10) for 1 h at 37°C. Virus inoculum was replaced with medium and the infection was carried for indicated times. The percentage of infected cells expressing GFP was measured by FACS.

In our studies, we used a recombinant RSV strain called rgRSV that expresses GFP [11] enabling us to quantify infection by FACS. The virus was grown in HEp-2 cells, and to minimize exposure to broken cells, harvested from the cell supernatant before cytopathic effects were observed. The quality of virus purified by gradient centrifugation was confirmed by SDS-PAGE (Fig. 1A).

When the purified particles were examined by indirect immunofluorescence (IIF) using antibodies to the F and the N proteins, we found three different particle populations. Half of the particles represented intact virions because in addition to F (green) they contained N (red) (Fig. 1B). Of these, 30% also stained with phalloidin (blue, pseudocolored white) indicating the presence of actin filaments as previously reported (Fig. 1B arrowheads) [12]. The remaining particles constituted capsid-free envelopes (VLPs). They stained for F but not for N. Since we did not detect free capsids that would stain only for N or P (data not shown), we used the presence of the capsid antigens to distinguish between intact RSVs and VLPs.

When purified virus preparations were incubated with HeLa cells at 4°C, immunoblotting after SDS-PAGE showed that more than half of the input N and P associated with the cells indicating that RSV binding in the cold was efficient (Fig. 1C).

To measure infection, RSV was added to HeLa cells for 1 h and infection was continued for additional 3–8 h before measuring GFP expression by FACS (Fig. 1D). The fraction of cells expressing GFP increased with time and with increasing multiplicity of infection (moi). In cells infected at moi of 10, GFP expression was detected as early as 3 h post-infection (hpi) (28% GFP positive cells) and after 5 hpi 80% of the cells were infected. At a moi of 3, GFP expression was delayed by about 3 h.

### RSV is endocytosed

**Figure 2:**
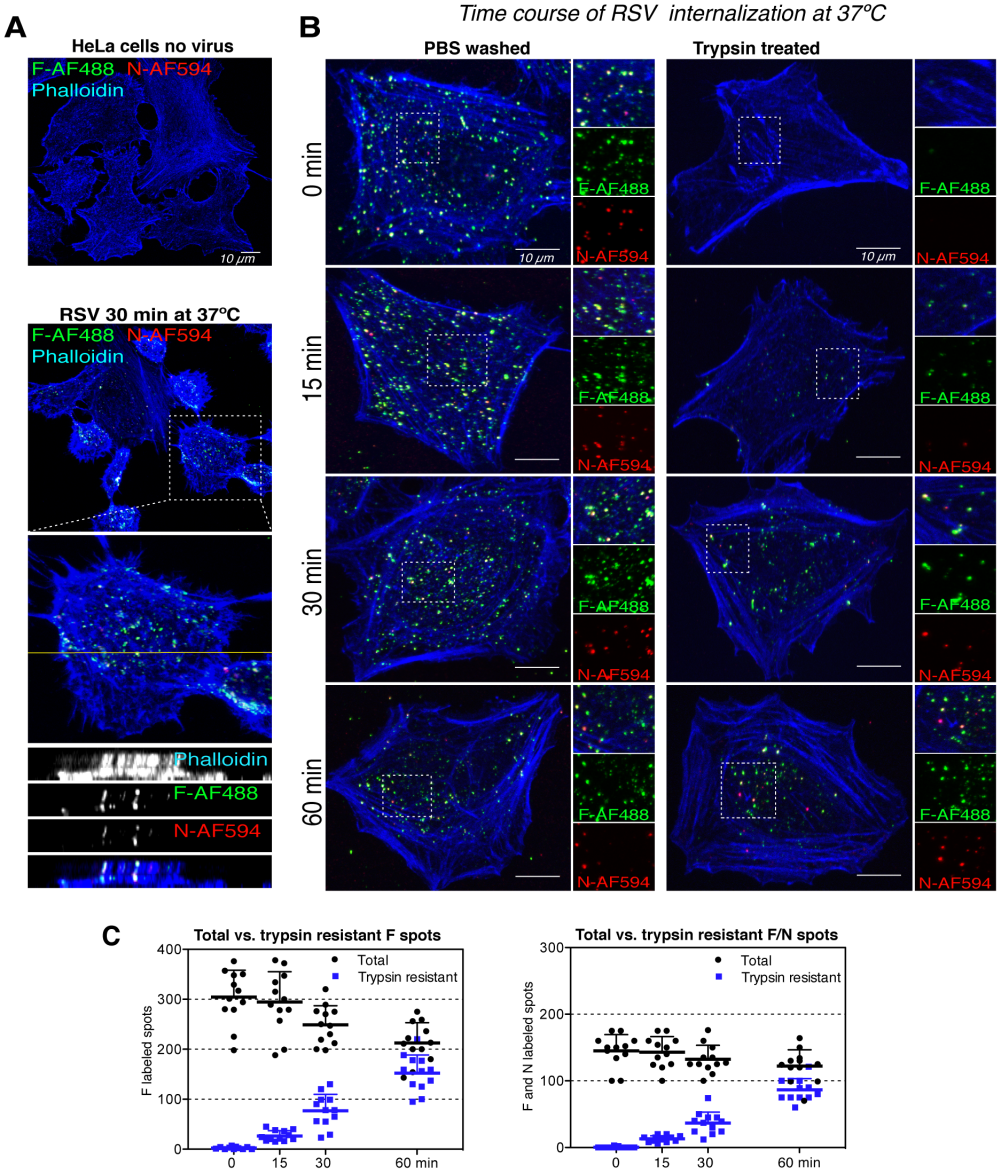
RSV undergoes endocytosis. (A). HeLa cells without (top) or with bound RSV (moi ∼0.5) at 4°C (bottom) were transferred for 30 min to 37°C before fixation. Cells were processed for confocal microscopy with anti-F-AF488 (green), anti-N-AF594 (red), and phalloidin-AF647 (blue), and Z-stack image series acquired. The orthogonal views of Z-stack projections (pseudocolored white) were generated with ImageJ. (B). RSV (moi ∼1) was bound to HeLa cells at 4°C, unbound virus was removed and cells transferred to 37°C. At indicated times cells were placed on ice and treated with PBS or trypsin for 3 min (PBS wash vs. trypsin treated). Samples were processed for confocal microscopy as in (A). (C). Quantification of the image series represented in (B). Analysis was performed with Imaris to detect spots with anti-F staining only (left) or anti-F and -N staining (right) at different times after warming the cells to 37°C.

To follow the fate of the cell-bound particles in the cold after warming to 37°C, IIF with anti-F and anti-N antibodies was used. Actin filaments were labeled with phalloidin to visualize cell boundaries. Confocal Z-stack image series in the orthogonal view revealed that after 30 min virus particles containing N and F were present not only on the cell surface but also deep inside the cytoplasm (Fig. 2A). This indicated that viral particles and VLPs were endocytosed.

After binding in the cold, cell-associated viruses and VLPs can be removed from the cell surface by brief incubation with trypsin in the cold that does not affect cell attachment (Fig. 2B, 0 min) [10]. We found that when cells after virus binding in the cold were incubated at 37°C, an increasing fraction of the cell-associated particles became trypsin resistant (Fig. 2B, 15–60 min). Quantitation using spot detection software Imaris showed that after 60 min, 77% VLP and 70% RSV-containing spots were, in fact, resistant to trypsin (Fig. 2C). That the total number of RSV- and VLP-containing spots decreased over time was probably caused by the accumulation of multiple particles in common endocytic vacuoles that represented single spots. Of the anti-F stained spots, 47% stained for N indicating that they were intact viruses.

### RSV endocytosis is followed by delayed intracellular fusion

To confirm that RSV was endocytosed in an intact form, it was important to determine whether the endocytosed particles also contained the viral lipid envelope. Purified RSV was therefore labeled with a lipophilic fluorescent dye, DiOC, which partitions into the viral membrane. It is fixable with formaldehyde, and can be quenched by the membrane-impermeable dye, trypan blue (TB) [13]. After labeling, 80% of re-purified particles contained detectable DiOC (data not shown).

**Figure 3:**
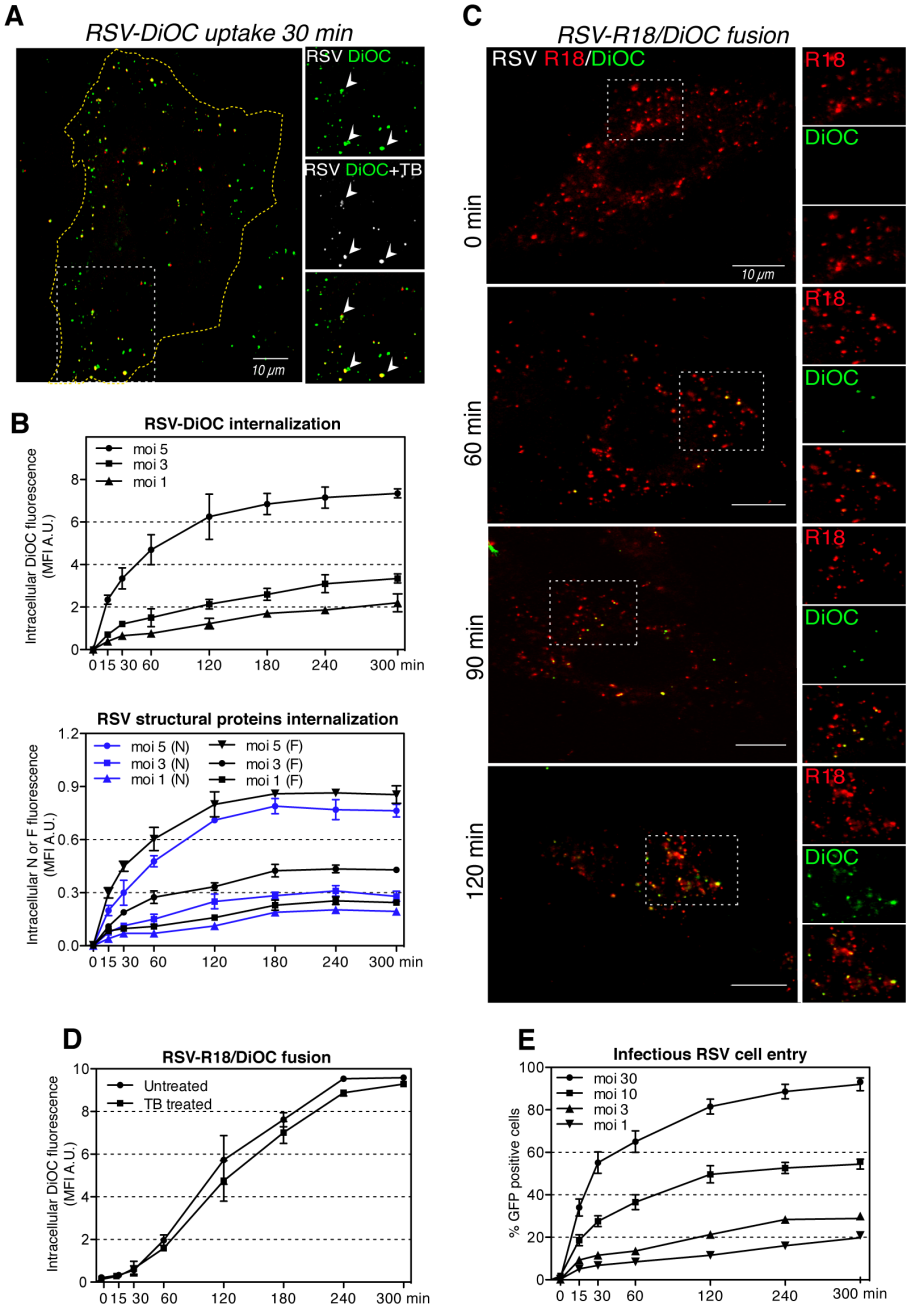
Rapid RSV endocytosis is followed by the intracellular fusion. (A). HeLa cells were incubated with RSV-DiOC (moi ∼3) for 30 min at 37°C. Single focal plane image of live cell samples was acquired with a confocal microscope before (green) and after TB addition (pseudocolored red/white). Arrowheads indicate virus spots that were not quenched after TB addition. (B). RSV-DiOC (moi ∼1–5) was bound to versene detached HeLa cells at 4°C for 1 h, unbound virus was washed away and cells were incubation at 37°C. (top) At the indicated times, cells were fixed and the mean fluorescence intensity (MFI) of DiOC measured by FACS in the presence of TB. (bottom) In parallel experiments at indicated times cells were trypsinized on ice for 10 min, washed, permeabilized, stained with anti-N-AF647 or anti-F-AF647 and the MFI of AF647 measured by FACS. (C). RSV-R18/DiOC (moi ∼5) was bound to HeLa cells at 4°C, unbound virus was removed and cells were incubated at 37°C. At indicated times, single focal planes of live cell samples were acquired with confocal microscope. (D). RSV-R18/DiOC (moi ∼5) was bound to versene detached HeLa cells at 4°C, unbound virus was washed away and cells were incubated at 37°C. At indicated times cells were fixed and the MFI of DiOC measured by FACS with or without TB. (E). RSV (moi ∼1 to 30) was bound to HeLa cells at 4°C for 1 h, unbound viruses were removed, and cells warmed to 37°C. At indicated times, samples were placed on ice and trypsinized for 10 min to remove virus remaining on the cell surface. Cells were washed and re-plated for up to 10 h before the percent of GFP expressing cells determined by FACS.

When added to cells and incubated at 37°C, the RSV-DiOC particles were visible as discrete fluorescent spots, and of these some were quenched when TB was added (Fig. 3A). FACS analysis showed that, 50% of the fluorescence was resistant to TB after 30 min, and full resistance was reached in about 180 min (Fig. 3B top), indicating that internalization of RSV and VLPs was rapid and complete. Importantly, when the intracellular accumulation of F and N proteins was measured in parallel (Fig. 3B bottom), internalization of both antigens and DiOC (Fig. 3B top) followed similar kinetics.

To monitor fusion of RSV with cellular membranes, we used a method developed by Sakai and coworkers [14]. In this case, RSV was labeled with two fluorescent lipids, R18 (red) and DiOC (green). Concentrations were used at which the R18 quenches the fluorescence signal emitted by the DiOC. Therefore, when allowed to bind to cells and viewed live by confocal microscopy, the labeled viruses were initially all red (Fig. 3C, 0 min). However, after about 60 min at 37°C, yellow and green intracellular spots became apparent increasing in numbers over time, because after fusion, the two lipids were diluted out and the green fluorescence of DiOC was no longer quenched by R18 (Fig. 3C). Some of the spots showed a ring-like fluorescence indicating that the DiOC was localized in the limiting membrane of intracellular vacuoles.

Quantitative FACS analysis showed that the dequenching of DiOC became detectable already after 30 min at 37°C (Fig. 3D). It reached a half maximal level at 90 min, and plateaued after 240 min. Treatment of cells with TB during FACS analysis revealed that more than 90% of the fluorescent DiOC failed to be quenched by this membrane impermeable agent confirming that the DiOC was localized in intracellular organelles. From the time course, it was apparent that the fusion events occurred on the average 50 min after endocytosis.

Our interpretation of these results was that the virus particles and VLPs that bound to the cell surface were endocytosed. Endocytosis was rapid and efficient, and the internalized viruses accumulated in endocytic vacuoles. After a lag period, the viral envelopes underwent fusion with vacuolar membranes.

To bring infection into the picture, cells with virus bound in cold were transferred to 37°C. At indicated times, they were placed on ice, incubated with trypsin to strip away surface-attached RSV, re-plated and incubated for 10 additional hours to allow infection to proceed and GFP to be expressed. FACS analysis demonstrated that in cultures that had been incubated at 37°C before trypsinization, the fraction of GFP-expressing cells increased with time. Maximum infection was reached within 180 min, and the half time was around 30 min (Fig. 3E). That the time course coincided perfectly with the time course of virus endocytosis (Fig. 3B) implied that productive infection depended on endocytosis.

### Endocytosis and infection are clathrin-, dynamin- and pH-independent

If RSV entry and infection depended on endocytosis as indicated by our experiments, we expected perturbants that inhibit endocytosis to block internalization and infection. In the experiments that followed, endocytosis of RSV was quantified by measuring the amount of the incoming N protein that was trypsin resistant using FACS analysis 1 h after warming. Infection was scored as a percentage of cells expressing GFP 6 hpi. It is important to mention that the dose-dependence (Fig. S1) and toxicity (data not shown) of each inhibitor was carefully determined. For clarity for most of the inhibitors we will present data at a single concentration where we found a strong effect but low cytotoxicity.

**Figure 4:**
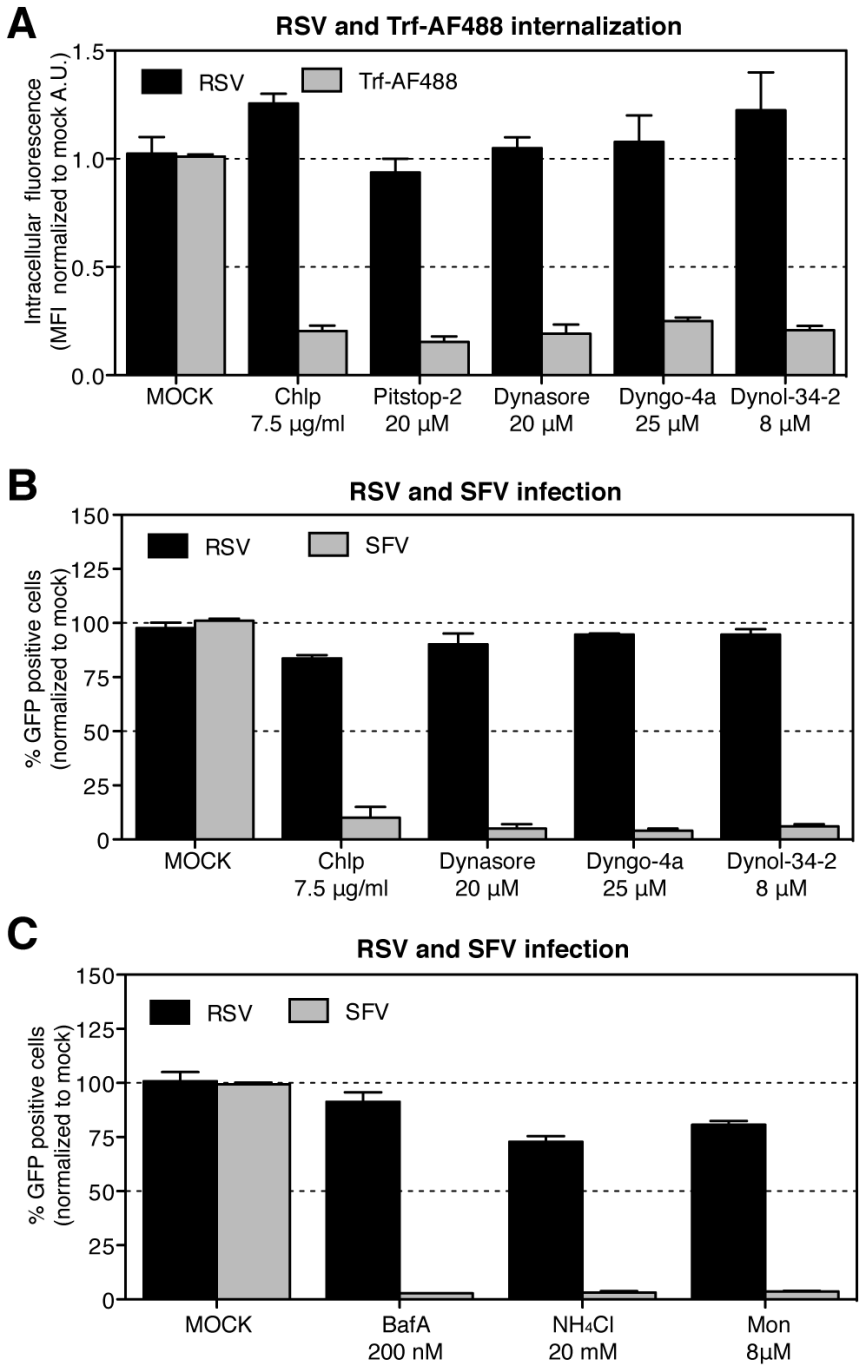
RSV endocytosis and infection are clathrin-, dynamin- and pH-independent. In A and B, HeLa cells were pretreated with solvent (MOCK), chlorpromazine (Chlp), pitstop-2, dynasore, dyngo-4a or dynol-34-2 at indicated concentrations and each inhibitor was continuously present during following steps of the experiment: (A) RSV (moi ∼3) or transferrin AF-488 (Trf-AF488) (2 µg/ml) were bound to cells at 4°C. After internalization at 37°C, RSV (1 h) or Trf-AF488 (20 min) cells were treated with trypsin on ice for 10 min before fixation. RSV infected cells were additionally stained by IIF with anti-N-AF488 antibody and the MFI of AF-488 measured by FACS. (B). Cells were infected with RSV (moi ∼3) or SFV-ZsGreen (moi ∼0.5) for up to 6 h before FACS analysis of GFP expressing cells. (C). HeLa cells were pretreated with bafilomycin A (BafA), ammonium chloride (NH_4_Cl), or monensin (Mon) at indicated concentrations and infected with RSV (moi ∼3) or ZsGreen-SFV (moi ∼0.5) in the continued presence of the compounds for up to 6 h before FACS analysis of GFP expressing cells.

Since RSV has been reported to enter cells by CME in HeLa cells [9], we tested five CME inhibitors: chlorpromazine [15], pitstop-2 [16], and three inhibitors of dynamin-2 (dynasore, dyngo-4a and dynole-34-2) [17]. None of them influenced RSV endocytosis, although, internalization of the well-characterized CME cargo protein transferrin (Trf) was efficiently inhibited by all (Fig. 4A). With the exception of pitstop-2, which was too toxic in the prolonged infection assay, none of the agents inhibited RSV infection (Fig. 4B). Infection by Semliki Forest virus (SFV), a virus known to depend on CME, was efficiently blocked by all (Fig. 4B) [18].

RSV infection has been reported to be insensitive to an increase in endosomal pH [9]. This was confirmed by the lack of influence of bafilomycin A, ammonium chloride, and monensin on RSV infection (Fig. 4C). As expected all three agents blocked infection by SFV, which needs low endosomal pH to trigger fusion [19]. The small reduction in RSV infection observed for ammonium chloride and monensin may reflect the importance of a balanced vacuolar environment for productive RSV infection.

Taken together, the results indicated that RSV endocytosis and infection did not depend on CME nor did it require acidification.

### Endocytosis is actin-dependent

**Figure 5:**
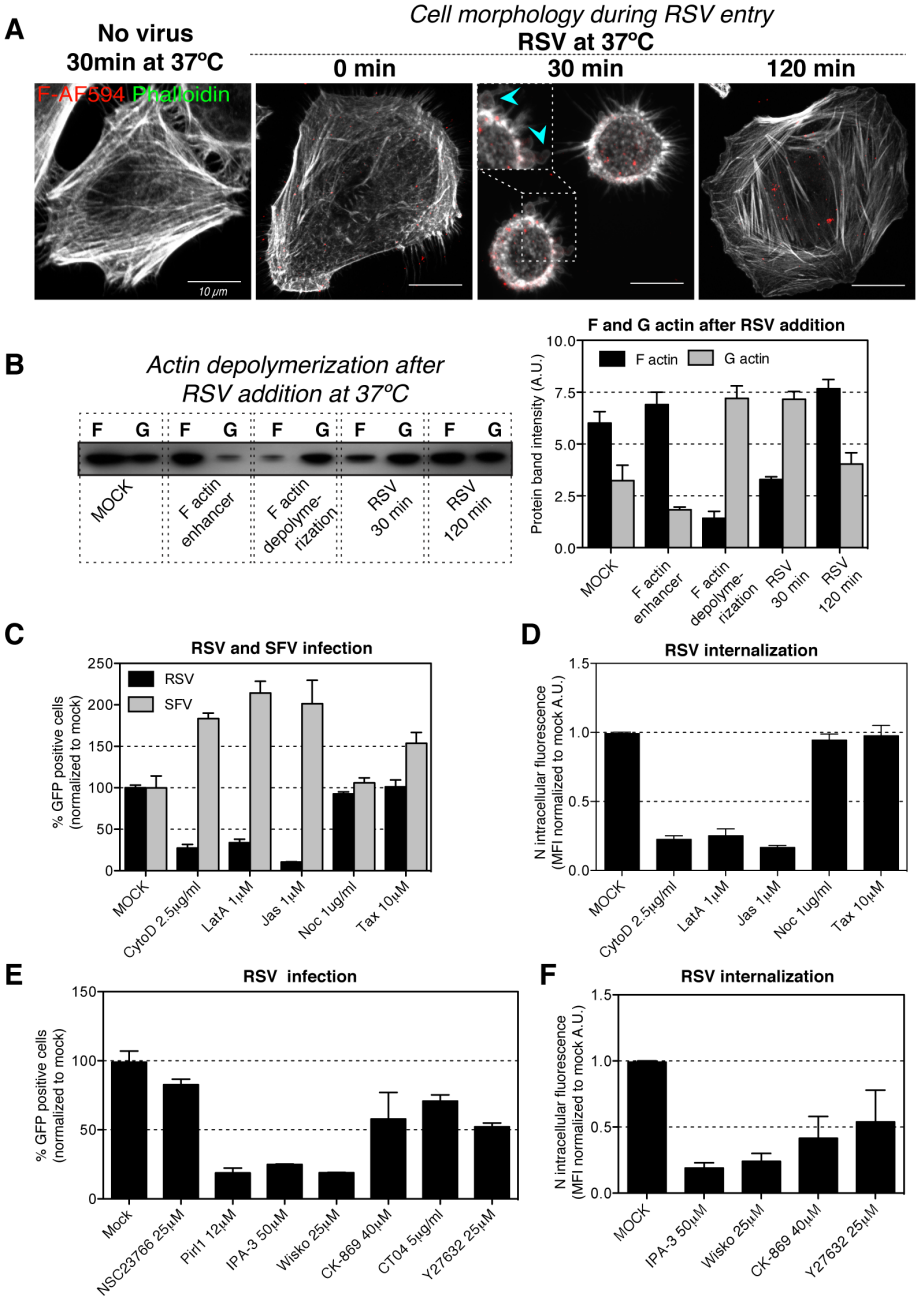
RSV infection induces actin rearrangement. (A). RSV (moi ∼0.5) was bound to HeLa cells at 4°C, unbound virus was removed and cells warmed to 37°C, fixed at indicated times, and stained with phalloidin-AF488 (pseudocolored white) and anti-F-AF647 (red) antibody. Images represent Z-stack projections acquired with a confocal microscope. Arrowheads show actin blebs formed at the cell surface. (B). RSV (moi ∼30) was incubated with HeLa cells for 30 or 120 min at 37°C. Samples were processed according to the kit manufacturer's protocol (Cytoskeleton Inc.). Controls included mock-treated cells and cells either treated with F actin enhancer or F actin depolymerizing agent. (left) The F and G actin fractions were resolved by SDS-PAGE and western blots probed with anti-actin antibody. (right) Quantification of actin protein bands intensities by densitometry. (C–F). HeLa cells were pretreated with solvent (MOCK), cytochalasin D (CytoD), latrunculin A (LatA), jasplakinolide (Jas), nocodazole (Noc), taxol (Tax), NCS23766, pirl1, IPA-3, wiskostatin (Wisko), CK-869, CT04, Y24632 at indicated concentrations and each inhibitor was continuously present during following steps of the experiment: (C, E). Cells where infected with RSV (moi ∼3) or SFV-ZsGreen (moi ∼0.5) for up to 6 hours before FACS analysis of GFP expressing cells. (D, F). RSV (moi ∼3) was bound to the cells at 4°C followed by 1 h of internalization at 37°C. Cells were trypsinized, fixed and stained with anti-N-AF488 antibody, and the MFI of AF-488 measured by FACS.

When RSV was bound to HeLa cells in the cold and the cells warmed to 37°C, rapid and dramatic changes in cell shape and actin distribution were observed (Fig. 5A). The number of actin stress fibers decreased, the cells rounded up, and transient blebs filled with actin formed on the cell surface (Fig. 5A, 30 min). These changes were clearly visualized by live cell imaging (Movie S1). The cell morphology and actin distribution returned to normal within 2 hpi. When the ratio of G (globular) and F (fibrous) actin was determined, it was found that 30 min after addition of RSV the ratio of G to F actin was 2∶1 compared to 1∶2 in control cells and in cells 2 h after virus addition (Fig. 5B). This indicated that addition of virus resulted in transient actin depolymerization.

Disruption of actin filaments with cytochalasin D and latrunculin A as well as filament stabilization by jasplakinolide were found to strongly reduce RSV infection, whereas SFV infection was enhanced (Fig. 5C). RSV endocytosis was also significantly decreased (Fig. 5D). Inhibition of Cdc42 (pirl1), inhibited RSV infection effectively, while inhibitors of Rac1 (NSC23766), RhoA (CT04) and its effector ROCK (Y27632) had only a moderate effect (Fig. 5E). Both infection and endocytic uptake were reduced when some of Cdc42's downstream effectors were inhibited including PAK1 (IPA-3), N-Wasp (wiskostatin), and moderately when Arp2/3 (CK-869) was targeted (Fig. 5E, F). Nocodazole and taxol that interfere with microtubules had no effect on RSV or SFV infection (Fig. 5D).

These results demonstrated that actin and its regulators played a critical role during RSV endocytosis and infection. F-actin was transiently depolymerized, resulting in the formation of blebs. In addition, Cdc42, PAK1, and N-Wasp were required for RSV internalization and infection.

### RSV induces fluid phase uptake

**Figure 6:**
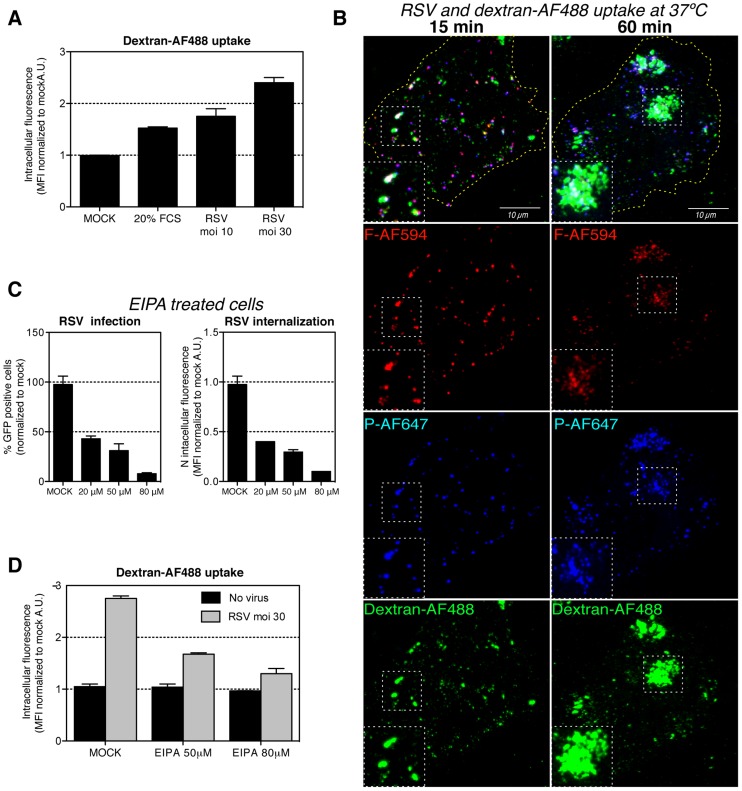
RSV induces bulk fluid phase uptake. (A). Serum starved HeLa cells were incubated with 20% FCS or purified RSV (moi ∼10, 30), at 4°C for 1 h. The inoculum was replaced with medium containing 10 kDa dextran-AF488 and transferred for 15 min to 37°C before fixation. The MFI of AF-488 measured by FACS. (B). Purified RSV (moi ∼10) was bound at 4°C to serum-starved HeLa cells. The input virus was replaced with medium containing 10 kDa dextran-AF488 (green) and transferred to 37°C. At indicated times cells were fixed, permeabilized and stained with anti-F-AF594 (red) and anti-P-AF647 (blue) antibody. Images represent a Z-stack projection acquired with the same confocal microscope settings. (C). HeLa cells were pretreated with solvent (MOCK) or EIPA at indicated concentration. (left) Cells were infected with RSV (moi ∼3) at 37°C for 6 hours before FACS analysis of GFP expressing cells. (right) RSV (moi ∼3) was bound to the cells at 4°C followed by 1 h of internalization at 37°C. Cells were trypsinized, fixed and stained with anti-N-AF488 antibody, and the MFI of AF-488 measured by FACS. (D). Serum starved HeLa cells were pretreated with EIPA at indicated concentration and incubated with purified RSV (moi ∼30) or no virus control at 4°C. The inoculum was replaced with medium containing 10 kDa dextran-AF488 and EIPA and transferred for 15 min to 37°C. Cells were fixed and the MFI of AF-488 measured by FACS.

The formation of blebs, the involvement of actin, and the role of Cdc42 and PAK1 suggested that infectious entry of RSV occurred by macropinocytosis as recently shown for several other viruses [for reviews see [20], [21]]. One of the characteristic features of macropinocytosis is an elevation in the uptake of extracellular fluid [22]. Indeed, when serum-starved HeLa cells were exposed to RSV, we observed that the internalization of 10 kDa dextran-AF488, a soluble, fluorescent tracer added to the medium, increased by 50% and 120% at low and high moi, respectively, over mock treated cells (Fig. 6A). The elevation was significantly higher than in serum-stimulated cells. IIF showed that majority of endocytosed viruses stained by anti-F and -P antibodies were localized in large, dextran-AF488 filled, intracellular vacuoles (Fig. 6B).

Macropinosome formation requires Na^+^/H^+^ exchanger (NHE) activity to modulate Rho GTPases at the PM [23]. Inhibition of NHE by EIPA (an amiloride derivative) has become one of the diagnostic criteria for macropinocytosis. EIPA inhibited RSV internalization and infection by 90% (Fig. 6C). In addition, pretreatment of cells with EIPA blocked the increase in fluid phase uptake induced by RSV (Fig. 6D). Taken together, these results demonstrated that RSV induces macropinocytosis and uses it for virus endocytosis and infection.

### Cellular regulators of macropinocytosis

**Figure 7:**
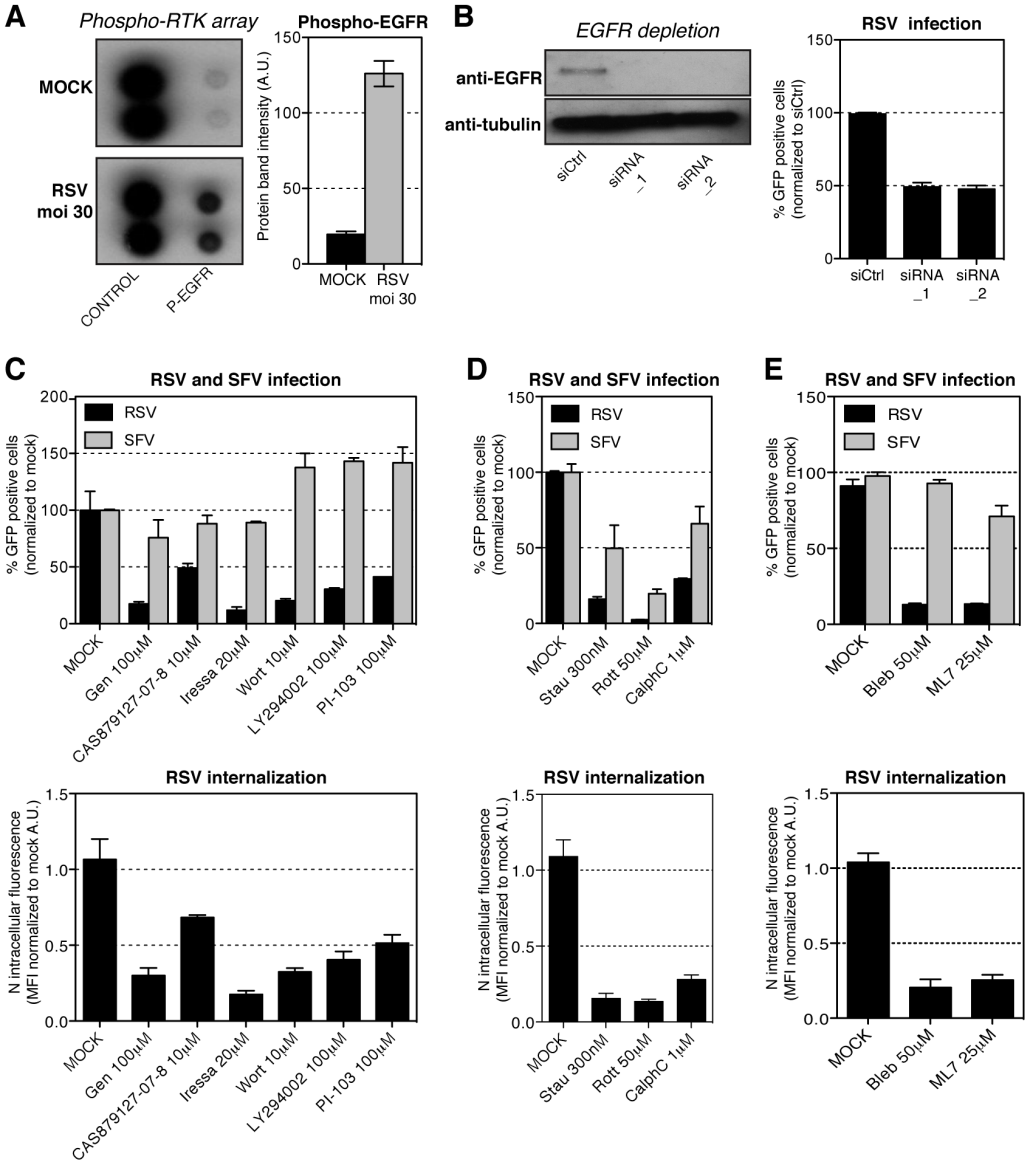
Cellular requirements for RSV endocytosis. (A). Purified RSV (moi ∼30) or mock that did not contain virus was added to serum-starved HeLa cells for 15 min at 37°C before cells were processed according to the kit manufacturer's protocol (R&D systems). (left) Representative array blots. (right) Quantification of phospho-EGFR signal intensity. (B). HeLa cells were revers transfected with scrambled siRNA (siCtrl) or siRNA against EGFR (siRNA_1, siRNA_2). After 72 hours cells were infected with RSV (moi ∼0.3) for 18 hours, before fixation and image based analysis. (left) Western blot validation of the siRNA EGFR depletion. (right) Image based quantification of RSV infection in cells with EGFR depletion. (C–E). HeLa cells were pretreated with solvent (MOCK) or (C) genistein (Gen), CAS879127-07-8, iressa, wortmannin (Wort), Ly294002 or PI-103 (D) staurosporine (Stau), rottlerin (Rott) or calphostine C (CalphC), (E) blebbistatin (Bleb), ML7 at indicated concentrations and each inhibitor was continuously present during following steps of the experiment. (C–E top) Cells were infected with RSV (moi ∼3) or ZsGreen-SFV (moi ∼0.5) for up to 6 hours, before FACS analysis of GFP expressing cells. (C–E bottom) RSV (moi ∼3) was bound to cells at 4°C followed by 1 h internalization at 37°C. Cells were trypsinized, fixed and stained with anti-N-AF488 antibody, and the MFI of AF-488 measured by FACS.

Macropinocytosis is usually initiated by activation of receptor tyrosine kinases (RTKs) or integrins, followed by the activation of a spectrum of cellular signaling factors [24]. Accordingly, we found that RSV infection was significantly decreased by two broad range protein kinase inhibitors; staurosporine (Ser/Thr kinases) and a multi-target protein tyrosine kinase inhibitor, genistein (Fig. 7C, D).

To test whether RTKs were involved, we used a human phospho-RTK array comprising antibodies against 42 different phosphorylated RTKs. Lysates from cells exposed to RSV for 15 min, and lysates from mock-treated control cells were used as probes. The epidermal growth factor receptor (EGFR) was the only RTK for which activation was detected; a five-fold increase in phosphorylation compared to control (Fig. 7A).

When the EGFR was depleted using siRNA, greater than 50% reduction in infection was observed (Fig. 7B). We found, moreover, that EGFR inhibitors (CAS879127-07-8, iressa) significantly decreased RSV infection (Fig. 7C). Inhibition of PI3K (wortmannin, Ly294002, PI-103), a downstream effector of EGFR, also reduced infection (Fig. 7C). EGFR inhibitors had little effect on SFV. That the PI3K inhibitors boosted SFV infection was consistent with a distinct entry mechanism for this virus. In addition, inhibition of PKC (rottlerin, calphostin C) decreased RSV (Fig. 7D). Although the effect on SFV was smaller, it suggested a role for PKC in the entry of both viruses. Finally, since non-muscle myosin II is thought to mediate closure of macropinosomes, we tested the effects of a myosin II inhibitor (blebbistatin), and a myosin light chain kinase inhibitor, (ML-7) [25]. Both reduced RSV infection with little effect on SFV (Fig. 7E).

All the inhibitors that decreased infection also reduced RSV endocytosis (Fig. 7C–E bottom). Depending on the compound, RSV internalization was reduced by 60–90%. None of the inhibitors affected RSV cell binding (Fig. S2). Thus, we concluded that infectious RSV cell entry and endocytosis were associated with activation of EGFR and its downstream signaling partners including PI3K and PKC. Combined with the requirement for myosin II, these findings were consistent with productive RSV internalization by macropinocytosis.

In addition, we performed a series of experiments in A549 cells (Fig. S3). They revealed changes in actin morphology and polymerization after addition of RSV, and a role of EGFR, NHE, Cdc42, Pak1, and other factors similar to HeLa cells in RSV infection. That internalization and infection were clearly dependent on the same cellular processes and factors in A549 cells indicated that entry by macropinocytosis was not HeLa cell specific.

### Intracellular trafficking and role of Rabs

**Figure 8:**
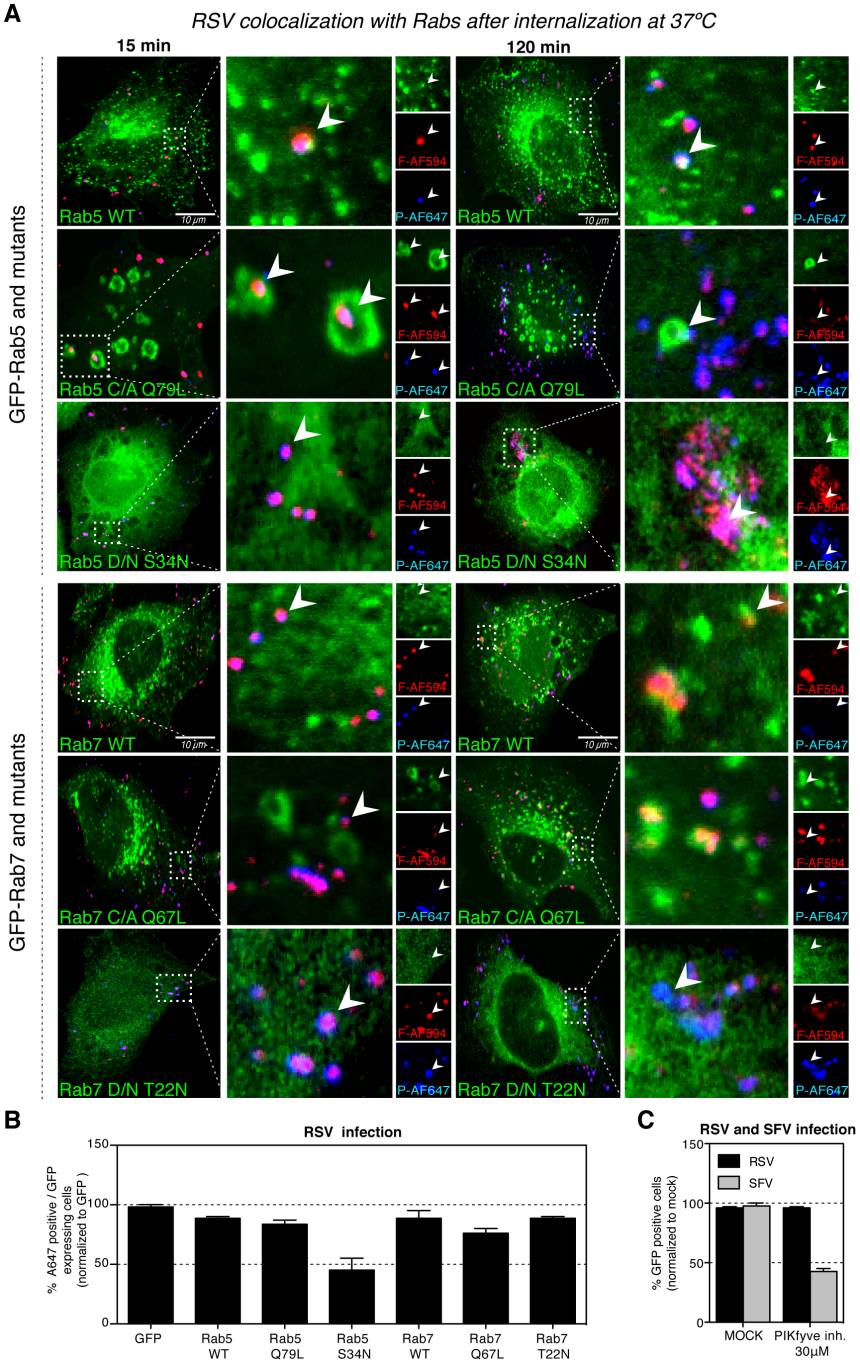
RSV virions traffic through Rab5 positive vesicles. (A–B). HeLa cells were transiently transfected with a GFP expressing constructs of Rab5 WT, -Rab5 Q79L (C/A), -Rab5 S34N (D/N), -Rab7 WT, -Rab7 Q67L (C/A), -Rab7 T22N (D/N) for 12 h. (A). RSV (moi ∼3) was bound to HeLa cells at 4°C, unbound virus was washed away and cells warmed at 37°C. At indicated time cells were fixed and stained with anti-F-AF594 (red) and anti-P-AF647 (blue) antibody. Images represent a single 0.37 µm thick focal planes acquired with the same confocal microscope settings. Arrowheads point the discussed in text phenotype of RSV and Rab colocalization. (B). Cells were infected with RSV-A2 (moi ∼0.5) for 16 hours before fixation and staining with anti-N-AF647. The number of AF647 positive cells among the population of GFP expressing cells was measured FACS. (C). HeLa cells were pretreated with 30 µM PIKfyve inhibitor (CAS 371942-96-7) or a solvent control (MOCK) and infected with RSV (moi ∼3) or ZsGreen-SFV (moi ∼0.5) for up to 6 hours before FACS analysis of GFP expressing cells.

Intracellular trafficking of macropinosomes is not well characterized, but it has been shown that like endosomes, they acidify and acquire Rab5 followed by Rab7 before fusing with endolysosomes [26]. Wild type (WT) GFP- Rab5 and GFP-Rab7 as well as various constitutively active (C/A) and dominant negative (D/N) mutants of the Rabs were transiently expressed in HeLa cells. After 15 min post warming, we observed that some of the incoming RSV colocalized with GFP-Rab5 WT positive vacuoles (Fig. 8A). Colocalization was even more evident in cells expressing the C/A GFP-Rab5 mutant Q79L that exhibits enlarged Rab5-positive vacuoles that fail to undergo further maturation [27]. There was no detectable colocalization with Rab7 at this time. After 120 min, some colocalization with GFP-Rab5 WT was still observed. In cells expressing the D/N GFP-Rab5 S34N, we noted accumulation of F- and P-stained particles inside large vacuoles in the perinuclear space. Colocalization of RSV with GFP-Rab7 WT was also detected.

To determine whether Rab5 and Rab7 played a role in infection, we infected cells expressing GFP-tagged constructs of Rab5, Rab7, and their mutants with RSV-A2. To determine the fraction of infected cells among cells expressing GFP-tagged Rabs, we stained the cells with anti-N-AF647. FACS analysis revealed that the D/N -GFP Rab5 (S34N) was the only Rab construct that caused a significant decrease in RSV infection when overexpressed (Fig. 8B). We confirmed this result by imaging of rrRSV expressing red fluorescent protein (Fig. S4).

Together with our imaging data, these results indicated that RSV depends on Rab5 GTP for infection but does not require Rab7. Infectious penetration is thus likely to be determined during early stages of macropinosome maturation. It is noteworthy that expression of the C/A Rab5 (Q71L), which is known to generate enlarged Rab5-containing endosomes and prevent endosome maturation and trafficking to lysosomes [27], did not affect infection. Pretreatment of cells with PIKfyve inhibitor (CAS 371942-96-7) had no effect on the RSV infection while SFV infection was decreased by 50% (Fig. 8C). By generating PtdIns(3,5)P2, PIKfyve is involved in the maturation of endosomes and macropinosomes [28]. This suggested that full maturation of macropinosomes was not required for RSV.

These results demonstrated that after pinching off from the PM, macropinosomes containing RSV acquired Rab5 and later Rab7. Maturation of macropinosomes involving Rab5 was evidently a critical step in infection, whereas later stages in maturation coordinated by Rab7 and PIKfyve were not essential.

### Post-endocytic cleavage of F

**Figure 9:**
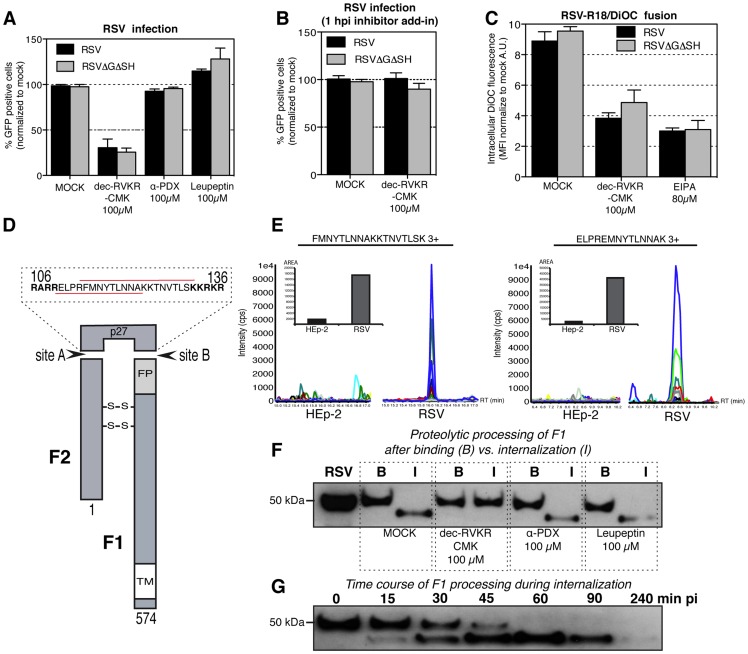
RSV F requires post endocytic activation. (A). HeLa cells were pretreated with dec-RVKR-CMK, α -PDX or leupeptin at indicated concentration for 1 h before experiment and inhibitors were continuously present during following steps of the experiment. Cells were infected with RSV or RSVΔGΔSH for 6 h before FACS analysis of GFP expressing cells. (B). HeLa cells were infected with RSV or RSVΔGΔSH for 1 h. Virus inoculum was replaced with medium containing 100 µM dec-RVKR-CMK and incubated for 6 before FACS analysis of GFP expressing cells. (C). Versene detached HeLa cells were pretreated with solvent (MOCK), dec-RVKR-CMK or EIPA at indicated concentrations and inhibitors were continuously present during following steps of the experiment. RSV-R18/DiOC or RSVΔGΔSH-R18/DiOC (moi ∼5) was bound to cells at 4°C. Unbound virus was removed and cells were incubated at 37°C for 2 h in the presence of inhibitor. Cells were fixed and the MFI of DiOC fluorescence measured by FACS and normalized to mock no inhibitor controls. (D). RSV F protein (574 aa) is proteolytically processed by furin like protease at the two sites (A aa-106 and B aa-136) to generate disulfide bonds linked F1+F2 and small peptide p27 (aa sequence depicted above). At the N-terminus of F1 is a FP (fusion peptide) and at the C-terminus TM (transmembrane domain), numbers indicate aa position and red underlines specify peptide sequences detected in mass spectrometry. (E). Proteomic analysis of HEp-2 cells and purified RSV particles. The N-terminal sequence of the p27 peptide (F protein) was quantified by a targeted mass spectrometry based on the selected reaction monitoring (SRM). Representative SRM peaks of peptides (left) FMNYTLNNAKKTNVTLSK 3+ and (right) ELPRFMNYTLNNAK 3+ peptides, corresponding to the aa 113–131 and 109–123 of F protein, respectively. Different SRM transitions for a peptide shown in different colors (see supporting information Table S1). The bar graphs show the results of the targeted peptide quantitation, presented as the sum of the areas of all the SRM peaks for a given peptide. Where no peptide peak was detectable, noise values were reported as a reference. RT retention time, and Cps counts per second. (F). HeLa cells were pretreated or MOCK treated with dec-RVKR-CMK, α-PDX, or leupeptin at indicated concentration. RSV (input control in the first line) was bound for 1 h in cold (B-binding) or after wash-away unbound virus was internalized for 1.5 h at 37°C before processing (I-internalized). Lysed cell samples were resolved by SDS-PAGE and blots were probed with anti-F1 antibody. (G). RSV was bound for 1 h in cold to HeLa cells; unbound virus wash away and cells were placed at 37°C for indicated times before, lysis, SDS-PAGE, and processing for western blot probed with anti-F1 antibody.

Since acidification of macropinosomes was not needed for infection, we speculated that RSV required some other intracellular cue to trigger fusion. The F is unique among paramyxovirus fusion proteins in having two cleavage sites for furin-like proteases generating in addition to F1 and F2 (50 and 20 kDa, respectively) a soluble 27 amino acid (aa) peptide (p27) [29], [30]. The p27 peptide is located between F2 and F1 N-terminal to the fusion peptide in F1 (Fig. 9D). We hypothesized that removal of this peptide after endocytosis might be required to activate the F protein.

Experiments in which cells were pretreated with a membrane permeable furin inhibitor, dec-RVKR-CMK, prior to addition of RSV indicated that a protease was indeed involved. Dec-RVKR-CMK treatment reduced infection by about 80% (Fig. 9A). That a membrane impermeable furin inhibitor, α-PDX, had no effect on infection suggested that the activating proteolysis did not occur on the cell surface. A broad range protease inhibitor leupeptin (serine, cysteine, threonine proteases) caused only a slight increase in infection.

That the inhibition of infection by dec-RVKR-CMK involved the F protein, was confirmed using a recombinant virus strain (RSVΔSHΔG) that lacks the SH and G glycoproteins [5]. Infection by this mutant virus was also blocked by dec-RVKR-CMK (Fig. 9A). When dec-RVKR-CMK inhibitor was applied 1 h after the virus inoculation, there was no inhibition indicating that the critical proteolytic step coincided with entry (Fig. 9B). Fusion assays with R18/DiOC labeled RSV and RSVΔSHΔG revealed that dec-RVKR-CMK impaired viral fusion (Fig. 9C). Its inhibitory effect was comparable to the effect of EIPA, which blocked virus internalization (Fig. 6C).

To confirm the presence of the p27 peptide in the purified virus, we used a targeted mass spectrometry approach based on selected reaction monitoring (SRM) (Fig. 9E). As a negative control, we analyzed HEp-2 cells extracts used to produce the virus. In trypsin digested virus preparations, we detected 2 peptides corresponding aa 113–131 and 109–123 of F protein both spanning p27 peptide (Fig. 9D). Neither peptide was present in HEp-2 control samples. SRM transitions of the targeted peptides are included as supporting information in Table S1.

Finally, SDS-PAGE was used to monitor changes in the F protein itself during entry. Blotting with an anti-F1 antibody revealed a protein band in the isolated virus migrating with a molecular weight of 50 kDa, confirming that the F protein had been cleaved at least once already in the producer cells (Fig. 9F). When virus bound to cells in the cold were allowed cell enter for 1.5 h at 37°C, the mobility of the cell-associated F1 became faster (48 kDa) indicating that further processing had occurred. The reduction in size of around 2 kDa was consistent with the loss of p27 at the N terminus of F1. Importantly, in cells pretreated with dec-RVKR-CMK, the processing of the 50 kDa F1 protein did not occur. As expected, α-PDX and leupeptin did not influence the processing step. When the time course of F1 processing was followed, we observed that some processed F1 was detectable already 15 min after cell warming (Fig. 9G). It peaked at 60 min when the precursor was fully consumed. That the amount of F1 protein gradually decreased at later time points was probably due to lysosomal degradation explaining the decrease in band intensity in the endocytosis lanes in Fig. 9F.

These results indicated that to become fusion competent and infectious, the F1 protein underwent a second, highly efficient cleavage by a furin-like convertase in an endocytic compartment. The time course indicated that the processing of F1 occurred soon after endocytosis preceding fusion by about 30 min.

### RSV enters polarized bronchial epithelial cells by macropinocytosis

**Figure 10:**
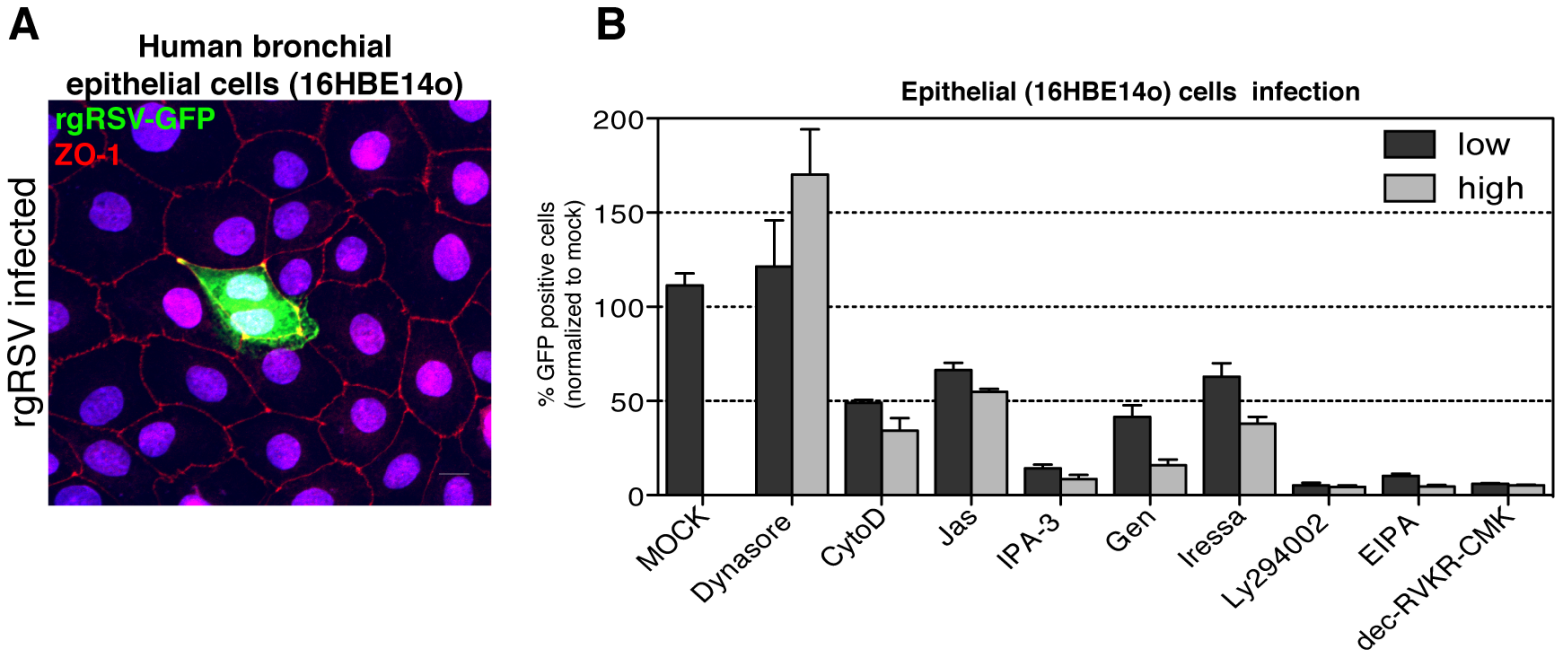
RSV enters human bronchial epithelial cells by macropinocytosis. (A). Human bronchial epithelial cells (16HBE14o) were polarized for 9 days and then infected with rgRSV for 20 h. After fixation cells were stained with anti-ZO-1-AF594 antibody (red) and TO-PRO-3 nuclear dye (blue) to examine polarization of the cell monolayer. RSV infection was visualized by GFP expression (green). (B) Polarized 16HBE14o cells were pretreated with solvent (MOCK), low or high concentrations of dynasore (10, 20 µM), cytochalasin D (CytoD 1.5, 3 µM), jasplakinolide (Jas 0.5, 1 µM), IPA-3 (40, 80 µM), genistein (Gen 50, 100 µM), iressa (10, 20 µM), Ly294002, EIPA (40, 80 µM), and dec-RVKR-CMK (50, 100 µM). The cells were infected with RSV for 20 h in the presence of the inhibitors, fixed and counterstained with DAPI. RSV infection was quantified by an image-based approach and normalized to mock infected controls.

To address whether our results applied to cell types infected by RSV *in vivo*, we tested polarized epithelial cells 16HBE14o obtained from human bronchial biopsies [31]. After 9 days in culture, the distribution of the tight junction marker, ZO-1, showed that the cells had reached a polarized phenotype (Fig. 10A). After making certain that RSV could infect 16HBE14o cells from the apical side (Fig. 10A), we tested the effects of nine diagnostic inhibitors previously used in HeLa cell experiments (Fig. 4B, 5CE, 6C, 7C, 9A). They inhibited dynamin, macropinocytosis, and furin proteases. RSV infection was quantified by an image-based approach that detected the fraction of GFP-expressing cells.

In agreement with our findings in HeLa cells, inhibition of dynamin by dynasore had no effect on RSV infection in 16HBE14o cells; it even boosted infection. EIPA and seven other inhibitors of macropinocytosis decreased infection in a dose-dependent fashion indicating that macropinocytosis was involved in entry. The need for F1 processing was confirmed by dec-RVKR-CMK, which was found to decrease infection by as much as 95%.

## Discussion

Paramyxoviruses are generally thought to infect cells by fusing directly with the PM [32], [33]. That paramyxovirus particles can also be endocytosed is, however, also clear. This has most recently been documented for Sendai, Nipah, RSV, Newcastle Disease viruses and for a lentivirus vector pseudotyped with measles virus glycoproteins [9], [34], [35], [36], [37], [38]. Which of the two pathways – fusion at PM or fusion after endocytosis - leads to infection is not clear.

In our experiments, we found that intact RSV was rapidly and efficiently endocytosed with capsid, glycoproteins, and the lipid envelope intact. The RSV and capsid-free VLPs accumulated within cytoplasmic vacuoles with a half time of about 30 min followed by fusion in the vacuoles with the half time of around 90 min. A sensitive fusion assay using R18/DiOC-labeled fluorescent viruses showed that fusion occurred intracellularly. No fusion of viruses with the PM was detected, and no formation of syncytia by fusion-from-without was observed even after exposing cells to high moi (data not shown). The significant delay between RSV internalization and fusion could at least in part be explained by the requirement for post-endocytic cleavage of F protein.

Perturbations that inhibited endocytic uptake caused a dramatic reduction in infection confirming a role for endocytosis in infection. EIPA inhibited both endocytosis and infection by 95%, and a similar level of inhibition was observed for agents that interfered with actin dynamics, a variety of kinases, and myosin II. The inhibitory effect of Rab5 D/N expression was also consistent with a role for endocytosis in infectious entry. Proteolytic activation of the F protein necessary for fusion and infection occurred in intracellular compartments. We concluded that RSV and VLPs were efficiently endocytosed, that penetration by membrane fusion occurred in endocytic vacuoles, and that at least 90% of infection was caused by endocytosed viruses.

That the endocytic mechanism responsible for the entry was macropinocytic was demonstrated by the following observations: (1) Strong dependence of endocytosis and infection on actin dynamics; (2) Transient activation of blebbing, loss of stress fibers, and cell shape changes after virus addition to cells; (3) Activation of EGFR phosphorylation and involvement of this receptor and its downstream signaling factors including PI3K, PKC, Cdc42, PAK1, and N-Wasp in virus endocytosis and infection; (4) Elevation of fluid uptake in the presence of virus, and the internalization of viruses together with fluid phase markers into large vacuoles; (5) Inhibition of endocytosis and infection by EIPA, an inhibitor of the NHE exchangers. Taken together, the observations satisfied all the main criteria currently used to define macropinocytosis [20], [21].

When inhibitor studies were performed using polarized physiologically relevant epithelial cells (human bronchial epithelium cells, 16HBE14o), infectious entry of RSV was found to depend on the actin cytoskeleton, on cell signaling, and on a furin-like protease activity as also observed in HeLa cells. The results indicated that infection of these polarized epithelial cells monolayers derived from human bronchial tissue involved macropinocytosis and proteolytic activation of the F protein.

Macropinocytosis is a clathrin-independent mechanism for the uptake of fluid and cell-associated particles within large, uncoated vesicles formed at the PM [24]. In most cell types, it is transiently induced by the activation of RTKs and downstream signaling factors [39]. In recent years, several viruses have been shown to use it for infectious cell entry. As recently reviewed [21], the best-described examples include large viruses such as vaccinia, Ebola, adeno 35, and Kaposi sarcoma-associated viruses. Interestingly, Nipah virus, a paramyxovirus of the Henipavirus subfamily, also belongs to this group. It uses EphrinB2A as a receptor, the phosphorylation of which is required for macropinocytic internalization and infection in CHO-K1 and VeroE6 cells [38].

We found that EGFR phosphorylation was activated by RSV, and that inhibitors such as iressa targeting this receptor blocked endocytosis and infection. It is noteworthy that iressa was only inhibitory when present during the first hour of virus cell contact confirming that its effect was entry-specific (data not shown). When the EGFR was depleted using siRNA, infection decreased only by 50% suggesting that other RTKs may be able to compensate in long-term experiments.

Downstream effectors of EGFR such as PI3K and PKC were also important for RSV endocytosis and infection. Infection has previously been shown to promote cell survival mediated by PI3K/NFκB. In A549 cells, PI3K activation and phosphorylation of its effector Akt occurs within 30 min after RSV addition [40]. Interestingly, it has also been demonstrated that RSV binding to NHEB cells induces PKC-α phosphorylation and translocation to the PM, while inhibition of PKC-α, as confirmed here, blocks RSV uptake and infection [41].

Our results contradicted a previous report proposing that RSV entry in HeLa cells occurs by CME [9]. The authors based their interpretation on hits such as clathrin and associated proteins in a targeted siRNA silencing screen against factors involved in endocytosis. (The hits also included actin modulators such as PAK1 but their function in entry was not addressed). However, since the read-out was infection after 20 h, a role for CME in post-endocytic steps in the RSV infectious cycle could not be excluded. In our experiments, we did not observe inhibition of RSV endocytosis or infection by five different agents that block CME: chlorpromazine, dynasore, pitstop-2, dyngo-4a, and dynol-34-2. Importantly these agents efficiently inhibited SFV, a virus that enters via CME. That dynasore fails to inhibit RSV infection was also recently reported by others [10].

While macropinosomes are still poorly characterized, there is evidence that they undergo a maturation process similar to that of endosomes involving acidification, association with Rab5 and Rab7, and fusion with late endosomes or endolysosomes [42]. We noted that some of the vacuoles containing RSV were in fact Rab5- and later Rab7-positive. Over-expression of a D/N Rab5 mutant inhibited infection suggesting that RSV penetration required passage through ‘early’ macropinosomes that contained Rab5. The lack of inhibition by Rab7 mutants, a PIKfyve inhibitor, and nocodazole, all known to inhibit vacuolar maturation, implied that macropinosome maturation beyond the Rab5 positive stages was not necessary.

Finally, our results provided a likely molecular explanation for the endocytosis requirement exhibited by RSV. Unlike other paramyxoviruses, the F protein in RSV has two activating cleavage sites [29]. Our mass spectroscopy analysis and western blots showed that while F in the isolated virus had been cleaved in the A-site (RARR) generating F2 and F1, it had not been cleaved at the more C-terminal B site (KKRKR). The second cleavage occurred after endocytosis. Inhibition of the second cleavage by dec-RVKR-CMK inhibited RSV fusion and infection. That dec-RVKR-CMK is a furin inhibitor suggested that the protease in question belonged to the furin family of convertases. The enzyme was evidently acid-independent, and active in early Rab5 –positive macropinosomes. Cleavage at the B site was most likely important because after removal the p27 peptide ‘cap’ from the N-terminus, the hydrophobic fusion peptide is rendered the most N-terminal sequence in F1. In other class I viral fusion proteins, including other paramyxovirus F proteins, the fusion sequence is invariably N-terminal [43].

In conclusion, we confirmed that RSV requires two cleavages in its F protein for infectivity and showed that the second cleavage occurs during cells entry. Infectious entry depends on endocytosis, which the virus induces by transiently activating macropinocytosis. The virus most likely meets the enzyme that generates the second cleavage in Rab5 positive macropinosomes, and fusion occurs after some delay in these vacuoles. In this respect, the virus resembles Ebola and SARS viruses, the fusion proteins of which are also activated within endocytic vacuoles by proteases [44], [45]. It is interesting to note that the F of Nipah virus, which has a single monobasic cleavage site in its F, is activated after endocytic uptake by cathepsin [46], [47]. Inhibitors of cathepsins block infection, and cathepsin double knock-out cells are not infected. The infectious entry of other paramyxoviruses (and other viruses that have pH-independent membrane fusion) may thus be endocytosis-dependent and the mechanisms more complex than previously assumed.

For RSV, it will now be important to analyze the molecular features of the entry process in more detail, to identify the protease(s), and to determine whether the intracellular route is relevant also *in vivo*. Being inducible and highly regulated, the macropinocytic process may prove more amenable to inhibition than other endocytic mechanisms, and therefore more easily targeted by therapeutics.

## Materials and Methods

### Cells and viruses

HeLa, A549 and HEp-2 cells were obtained from the ATCC and cultured in DMEM supplemented with 10% fetal calf serum (FCS), 1 mM Hepes, 1% Glutamax (Invitrogen). Transformed bronchial epithelial 16HBE14o cells obtained from Dr. D. Gruenert [31], [48], were grown in RPMI 1640 medium supplemented with 10% FCS, 1% l-glutamine and 1% NEA for at least 9 days before infection.

Recombinant SFV-ZsGreen stocks were kindly provided by Dr. G. Balistreri [49]. RSV-A2 was purchased from ATCC. Recombinant RSV strains expressing GFP (rgRSV, rgRSVΔSHΔG) or RFP (rrRSV) were kindly provided by Drs. M. Peeples and P. Collins [11], [50]. RSV was produced in HEp-2 cells. Virus was collected form cell culture supernatant.

### Inhibitors, antibodies and plasmids

The inhibitors used included: PI-103 (Alexis Biochemicals), dynasore, dyngo-4a,dynol-31-2, pitstop-2 (Ascent Scientific), pirl1 (Chembridge), wiskostatin (Enzo), CAS 879127-07-8, CAS 371942-96-7, dec-RVKR-CMK, LY294002, NSC23766, staurosporine, taxol, wortmannin, Y27632, α-PDX (Calbiochem), bafilomycin A, blebbistatin, calphostin C, chlorpromazine, cytochalasin D, EIPA, genistein, IPA-3, jasplakinolide, latrunculin A, leupeptin, ML7, monensin, NH4Cl, nocodazole, and rottlerin (Sigma).

Antibody and fluorescent dyes that were used comprised: anti-N monoclonal MAB858-3-5 and anti-RSV goat polyclonal AB1128 (Millipore), anti-F monoclonal ab43812 (abcam), anti-P rabbit polyclonal (3-V Biosciences, Menlo Park, USA), anti-ZO-1, goat anti-mouse, goat anti-rabbit, donkey anti-goat AF-conjugated, 10 kDa dextran- AF488, phalloidin AF-conjugated, R18, DiOC, and transferrin-AF488 (Molecular Probes), goat anti-mouse, goat anti-rabbit HRP-conjugated (Bio-Rad).

Expression plasmids encoding GFP-tagged Rab5, Rab7 and its mutants were kindly provided by Dr. M. Zerial (Max Planck Institute, Dresden, Germany).

### Virus growth and purification

HEp-2 cells (50–60% confluent) in T175 flasks were infected with RSV (moi 0.1) in 8 ml serum free DMEM-Hepes medium (DMEM, 1 mM Hepes) for 1 h at 37°C, and then inoculum was replaced with complete medium (DMEM, 10% FCS, 1% Glutamax, 1 mM Hepes). After 48 h, the cell supernatant was collected, clarified by centrifugation, aliquoted, snap frozen, and stored in −80°C for experiments that did not require purified virus.

For virus purification the method of Ueba [51] was modified as follows. The pre-cleared cell supernatant was centrifuged (20.000 rpm, 90 min, 4°C, SW32 Ti rotor, Beckman Optima 90-K ultracentrifuge) through 8 ml 20% w/v sucrose cushion in HBSS-Hepes buffer (HBSS, 25 mM Hepes). Pellets were gently washed, reconstituted in 20% sucrose and centrifuged in a 35, 45, 60% sucrose step gradient (35.000 rpm, 90 min, 4°C, SW41 rotor, Beckman Optima 90-K ultracentrifuge). An opalescent virus band was collected from the 35 and 45% interface, overlaid over a 30–60% continuous sucrose gradient and centrifuged (35.000 rpm, 4 h, 4°C, SW41 rotor, Beckman Optima 90-K ultracentrifuge). The virus fraction at about 45% sucrose was harvested, diluted in the HBSS-Hepes and pelleted by additional centrifugation (20.000 rpm, 90 min, 4°C SW41 rotor, Beckman Optima 90-K ultracentrifuge). Virus pellets were resuspended in HBSS-Hepes, snap frozen, and stored in −80°C.

All RSV stocks were titered by infecting HEp-2 cells with serial dilutions of the virus in 96 well plates. Infection was allowed to proceed for 18–22 h at 37°C. Fixed cells were assessed by microscopy for GFP expression, or stained with RSV anti-N antibody (AF-488) to detect infected cells of rgRSV or RSV-A2 respectively. Protein assay (Bio-Rad) was used to measure the amount of protein.

### Pharmacological inhibitors treatment

When the influence of pharmacological inhibitors was tested, cells were preincubated with a medium containing inhibitors for 30 min at 37°C before virus binding or infection (except Rho GTPase and protease inhibitors that required 1–5 h of preincubation, and pitstop-2 which was preincubated for 10 min). Inhibitory compounds at indicated concentration were continuously present during all of the steps of infection and internalization experiments.

### RSV DiOC labeling and endocytosis assay

DiOC (6 µg) was added to freshly purified RSV (25 µg) in 1 ml of HBSS-Hepes buffer and incubated at room temperature while gently shaking for 1 h. To remove unincorporated dye, virus was filtered through 0.45 µm syringe filter (Millipore) and snap frozen as 100 µl aliquots, stored in −80°C, and used within 2 weeks. Labeled RSV-DiOC was titered in HEp-2 cells as described above.

For the RSV-DiOC endocytosis assay, cells were detached with versene solution (0.53 mM EDTA, pH 8.0), washed with PBS and chilled on ice. Purified RSV-DiOC, diluted in DMEM-Hepes was bound to the cells on ice for 1 h. The inoculum was washed away with PBS; the cells were resuspended in complete medium and transferred to 37°C. After desired times, the cells were fixed in 4% formaldehyde, washed, and resuspended in FACS buffer (PBS, 0.2% FCS, 5 mM EDTA). For the FACS data acquisition (BD Biosciences, Canto II) samples were divided into two. One sample was acquired directly and the other after addition of trypan blue to a final concentration of 0.01% (Invitrogen).

Alternatively, after internalization cells were treated with 0.5% trypsin for 10 min on ice. Than washed with PBS, fixed in 4% formaldehyde, permeabilized with 0.01% Triton X-100, stained with anti-F or anti-N antibody at 4°C over night followed by the AF-647 secondary antibody staining. Mean fluorescence intensity measured by flow cytometry (BD Biosciences, Canto II) was normalized to the mock control without bound virus.

### RSV R18/DiOC labeling and fusion assay

Purified RSV (25 µg) was resuspended in 0.5 ml of HBSS-Hepes buffer before adding of DiOC (3.3 µM) and R18 (6.7 µM) mixture. Labeling was performed for 1 h at room temperature while gently shaking. To remove unincorporated dye, virus was filtered through 0.45 µm syringe filter (Millipore) and used freshly for the fusion assay.

For the RSV-R18/DiOC fusion assay, cells were detached with versene solution, washed with PBS and chilled on ice. Purified RSV-R18/DiOC, diluted in DMEM-Hepes was bound to the cells on ice for 1 h. The inoculum was washed away with PBS; the cells were resuspended in complete medium and transferred to 37°C. After desired times, the cells were fixed in 4% formaldehyde, washed, and resuspended in FACS buffer. For the FACS data acquisition (BD Biosciences, Canto II) samples were divided into two. One sample was analyzed directly and the other after addition of trypan blue to a final concentration of 0.01% (Invitrogen).

### FACS based RSV infection assay

Subconfluent cells were infected with RSV (moi ∼3) for 1 h at 37°C. Unbound virus was washed with PBS, cells were supplemented with complete medium and infection carried for 6 h. Then, cells were trypsinized, fixed in 4% formaldehyde, resuspended in FACS buffer. GFP expression determined by FACS (BD Bioscience, Canto II) and analyzed by FlowJo 9.1. Results of the infection in the presence of tested compounds were normalized to the solvent treated control cells. Experiments with ZsGreen-SFV infection were performed in parallel under identical conditions, with the exception that cells were harvested after 4 h.

### FACS based RSV internalization assay

RSV (moi ∼3) was bound to subconfluent cells for 1 h at 4°C. Than cells were washed with PBS, supplemented with medium containing 1 mM cycloheximide and incubated for 1 h at 37°C. Cells were moved on ice and treated with ice cold 0.5% trypsin for 10–20 min, washed with PBS, fixed in 4% formaldehyde, permeabilized with 0.1% Triton X-100 and stained with anti-N antibody over night at 4°C followed by the goat anti-mouse AF staining. The MFI (mean fluorescence intensity) of AF staining determined by FACS (BD Bioscience, Canto II) and analyzed by FlowJo 9.1. Results were normalized to the solvent treated control cells and presented as arbitrary units (A.U.).

For the Trf-AF488 (2 µg/ml) internalization was performed in parallel in identical conditions with the exception that uptake was carried for 20 min.

### FACS based RSV binding assay

Adherent cells were detached with versene solution, washed with PBS, chilled on ice, and pelleted. Cell pellets were resuspended in DMEM-Hepes, containing the virus inoculum, and incubated 1 h at 4°C. When the effect of inhibitors was tested, cells were preincubated with media containing inhibitor for 30 min, and inhibitor was continuously present at each step of the experiments. Cells were washed with PBS to remove unbound virus, fixed with 4% formaldehyde, permeabilized with 0.01% Triton X-100, and stained with RSV anti-F over night at 4°C, followed by the secondary antibody labeling. Samples were reconstituted in FACS buffer and analyzed by flow cytometry (BD Biosciences, Canto II). Mean fluorescence intensity values were normalized to the mock control without bound virus.

### Quantification of the RSV binding by western blot

Cells were detached with versene solution, washed with PBS, chilled on ice, and pelleted. Virus input (purified virus stock (moi ∼10) diluted in DMEM-Hepes) was divided in two portions. One was mixed with the cells and the other left for further analysis as virus input. Virus was incubated with the cells for 1 h at 4°C. The cells were pelleted by centrifugation and the supernatant collected as an unbound viruses sample. The cell pellets were washed in PBS and resuspended in DMEM-Hepes of equal volume to the collected supernatant. The SDS-PAGE loading buffer was added to all samples, followed by denaturation for 10 min at 95°C. An equal volume of each sample was separated by the SDS-PAGE and subjected to western blot with anti-P or anti-N antibody. Western blots were quantified by densitometry with QuantityOne software (Bio-Rad).

### Fluid phase uptake

Subconfluent cells seeded in 24 well plates were starved over night with serum free DMEM-Hepes medium. Purified RSV was bound to pre-chilled cells for 1 h at 4°C. Virus inoculum was washed and cells were pulsed with the serum free DMEM-Hepes medium containing 10 kDa dextran-AF488 (0.5 mg/ml) for 15 or 60 min at 37°C. The cells were washed with 10 mM NaOAc, 50 mM NaCl pH 5.5 followed by PBS wash. The cells were trypsinized, fixed in 4% formaldehyde and analyzed by flow cytometry (BD Biosciences, Canto II). For confocal microscopy, cells were fixed in 4% formaldehyde, permeabilized with 0.1% Triton X-100, and stained with RSV specific antibodies. Images were acquired with Zeiss LSM510 laser scanning confocal microscope.

### Transfection and transient expression

For transfection, cells were trypsinized, pelleted, and electroporated with 2 µg of plasmid DNA using Amaxa (Nucleofactor solution R, program I-13). Cells were seeded on 12 mm cover glass in 24 well plates for imaging or in the 6 well plates for FACS analysis. Experiments were performed after 12 h of transient expression.

### F/G actin extraction

The experiment was performed according to the protocol provided by the F/G actin *in vitro* assay kit producer (Cytoskeleton Inc. cat # BK037). In brief, subconfluent cells in 3.5 cm dishes were inoculated with purified rgRSV (moi 30) for 30 or 120 min at 37°C. Cells were washed, lysed, and clarified by centrifugation. Supernatants were centrifuged (53000 rpm, 1 h, 37°C, TLA120.2 rotor, Beckman Optima TLX ultracentrifuge), the resulting supernatants were collected (G-actin), and the pellets (F-actin) were resuspended in equal to supernatant volume of water containing actin depolymerizing reagent provided in the kit. Equal volume of each sample was resolved by SDS-PAGE and western blots were developed with an anti-actin antibody. To measure ratio of the G and F actin western blots were analyzed by densitometry with QuantityOne software (Bio-Rad).

### siRNA transfection and infection analysis

For the siRNA experiment, 3000 cells were revers transfected with Lipofectamine RNAiMAX (Invitrogen) with siRNA (siCtrl scrambled, siRNA_1 ATAGGTATTGGTGAATTTAAA, siRNA_2 AAGCTCACGCAGTTGGGCACT, NM_005228, NM_201282, NM_201283, NM_201284, Qiagen) in the optical bottom 96 well plates. Cells were infected with rgRSV (moi 0.3) 72 h post transfection. After 18 h cells were fixed with 4% formaldehyde and counterstained with DAPI.

### Image-based infection analysis

For the infection assays cells were plated in optical bottom 96 well plates. After infection with rgRSV for 18–20 hours, the cells were fixed with 4% formaldehyde and counterstained with DAPI. Nine images per well were acquired with automated MD2 microscope that autofocuses at each image acquisition (10× objective). The total cells number and the number of infected cells expressing GFP were scored by the MatLab-based infection counter software described previously [52].

### Human phospho-RTK array

Human phospho-RTK array kit was obtained from R&D Systems (cat # ARY001) and experimental procedure was followed according the manufacturers guidelines. In brief, serum-starved subconfluent cells in 3.5 cm dish were inoculated with purified RSV (moi 30) or null virus prep extract for 15 min at 37°C. Then, cells were lysed and pre-clarified by centrifugation. The supernatant samples were incubated with the antibody array, and developed with provided reagents. Developed arrays were analyzed by densitometry with QuantityOne software (Bio-Rad).

### Detection of the F by western blot

Subconfluent cells in 12 well plates were chilled on ice for 20 min. RSV (moi 10) in DMEM-Hepes was bound to cells for 1 h at 4°C. Post binding samples were washed and lysed in RIPA buffer (50 mM Tris, 150 mM NaCl, 2 mM EDTA, 1% NP-40, 0.1% SDS, pH 7.4). Remaining samples were washed with PBS supplemented with complete medium and transferred to 37°C for 1 h; following cells were trypsinized, washed and lysed with RIPA buffer. All samples were separated on the 10% Bis-Tris gels (Invitrogen). Western blots were probed with anti-F antibody (1∶500), HRP conjugated goat ant-mouse (1∶2000) and developed with super signal west pico (Pierce).

### Immunofluorescence staining and analysis

0.8×10^5^ cells were seeded on 12 mm coverslips in the 24 well plate 24 h prior experiment. Plates were chilled on ice and then RSV was bound to cells in DMEM-Hepes for 1 h at 4°C. Viruses were washed away and cells were supplemented with complete medium and transferred to 37°C. At desired times cells were fixed in 4% formaldehyde, permeabilized with 0.1% triton X-100 and incubated with 10% goat serum for 30 min. Cells were then stained with appropriate primary and secondary antibody. Coverslips were mounted to the glass slides with prolong gold anti-fade reagent (Invitrogen).

### Microscopy

Immunofluorescence images were captured with LSM Zeiss 510 microscope with the confocal laser scanning set up (objectives 60 or 100×). Per experiment, 10–15 images were captured and processed by ImageJ. For the virus particles detection Imaris software was used set up to detect particles larger than 0.5 µm and quality greater than 15.

Live cell imaging was performed with Olympus CellR, 20× objective with DIC setting, equipped with 37°C incubator.

### Sample preparation for mass spectrometry

Hep2 cells or gradient purified RSV particles were lysed in denaturing buffer containing 8 M urea and 100 mM NH_4_HCO_3_. Lysates were briefly sonicated and proteins were reduced with 12 mM DTT for 30 min at 32°C and alkylated with 40 mM iodoacetamide for 45 min at 25°C. The samples were diluted 1∶5 with 100 mM NH_4_HCO_3_ and digested with sequencing-grade porcine trypsin (Promega) at an enzyme/substrate ratio of 1∶100. The digestion was performed overnight and stopped with formic acid at final concentration of 2%. The peptide mixtures were desalted on Sep-Pak C18 cartridges (Waters), eluted with 80% acetonitrile, vacuum centrifuged until dryness and resuspended in 0.15% formic acid.

For each peptide, Q1 and Q3 masses, as well as collision energies (CE) for peptide fragmentation were calculated using Skyline (v1.3, MacCoss Lab Software). Double and triple charged product ions from the y-and b-series and transitions in a mass range of 350–1250 Da were considered (see full list of SRM transitions in the supporting information Table S1). The peptide samples were measured in SRM mode on a triple-quadrupole/ion trap mass spectrometer (5500QTrap, ABSciex) equipped with a nano-electrospray ion source. For the chromatographic separation of the peptides, the instrument was coupled with an Eksigent Nano LC system (ABSciex) connected to a 15-cm fused silica column, 75 µm inner diameter (BGB Analytik), packed in-house with Magic C18 AQ, 5 mm beads (Michrom Bioresources). The peptide mixtures were loaded from an autosampler cooled to 4°C (ABSciex) and separated with a linear gradient of acetonitrile/water containing 0.1% formic acid from 5 to 35% acetonitrile in 30 min, with a flow rate of 350 nl/min. SRM analysis was conducted with Q1 and Q3 operated at unit resolution (0.7 m/z half maximum peak width) with up to 70 transitions per run (dwell time, 30 ms; cycle time, 2.5 s). Data were analyzed with the software Skyline. Peak area of the SRM peaks was used for quantitation, after confirming co-elution and shape similarity of the transitions monitored for each peptide. Outlier transitions (e.g., shouldered or noisy transition traces) were not considered in the calculations. Results are presented as the sum of the areas of all SRM peaks for a given peptide.

### Statistical analysis

All experiments were performed at least in triplicate, and presented as normalized values with +/− standard deviation (SD).

## Supporting Information

Figure S1 **Dose dependent influence of inhibitors on the RSV infection.** HeLa cells were pretreated with solvent (MOCK), or (A) chlorpromazine (Chlp), dynasore, dyngo-4a, dynol-34-2, bafilomycin A (BafA), ammonium chloride (NH4Cl), monensin (Mon) (B) cytochalasin D (CytoD), latrunculin A (LatA), jasplakinolide (Jas), nocodazole (Noc), taxol (Tax), NCS23766, pirl1, IPA-3, wiskostatin (Wisko), CK-869, CT04, Y24632 (C) genistein (Gen), CAS879127-07-8, iressa, wortmannin (Wort), LY294002, PI-103, staurosporine (Stau), rottlerin (Rott), calphostin C (CalphC), blebbistatin (Bleb), ML7, (D) dec-RVKR-CMK, PDX or Leupeptin at indicated concentrations and individual inhibitor were continuously present at each step of the experiment. (A–D). Cells were infected with RSV (moi ∼3) for 6 hours, before FACS analysis of GFP expressing cells.
(TIF)

Figure S2 **RSV cell binding to cells treated with inhibitors.** HeLa cells were pretreated with solvent (MOCK), genistein (Gen), CAS879127-07- 8, iressa, wortmannin (Wort), LY294002, PI-103, staurosporine (Stau), rottlerin (Rott), calphostin C (CalphC), blebbistatin (Bleb), ML7 at indicated concentrations and individual inhibitor were continuously present at each step of the experiment. Cells were chilled on ice and RSV (moi ∼3) was bound to the cells in cold for 1 h. Cells were fixed, permeabilized, stain with anti-F-AF488 antibody, and the MFI of AF-488 measured by FACS.
(TIF)

Figure S3 **RSV enters A549 cells by macropinocytosis.** (A). RSV (moi ∼0.5) was bound to A549 cells at 4°C followed by 30 min at 37°C. Cells were by IIF with anti-F-AF488 (green), anti-N-AF594 (red), and phalloidin- AF647 (pseudocolored white) for confocal microscopy as, and Z-stack image series acquired. The orthogonal views of image Z-stacks (pseudo-colored white) were generated with ImageJ. (B). RSV (moi ∼0.5) was bound to A549 cells at 4°C, virus inoculum was washed, cells warmed to 37°C, fixed at indicated times, and stained with phalloidin-AF488 (pseudo colored white) and anti-F-AF647 (red) antibody. Images represent Z-stack projections acquired with a confocal microscope. (C). RSV (moi ∼30) was incubated with A549 cells for 30 or 120 min at 37°C. Samples were processed according to the kit manufacturer's protocol (Cytoskeleton Inc.). Controls included mock-treated cells and cells either treated with F actin enhancer or F actin depolymerizing agent. (left) The F and G actin fractions were resolved by SDS-PAGE and western blots probed with anti-actin antibody. (right) Quantification of actin protein bands intensities by densitometry. A549 cells were pretreated with solvent (MOCK) or (D–E) cytochalasin D (CytoD), latrunculin A (LatA), jasplakinolide (Jas), nocodazole (Noc), taxol (Tax), (F) NCS23766, pirl1, IPA-3, wiskostatin (Wisko), CK-869, CT04, Y24632, (G) genistein (Gen), CAS879127-07-8, Iressa, wortmannin (Wort), LY294002, PI-103, staurosporine (Stau), rottlerin (Rott), calphostin C (CalphC), blebbistatin (Bleb), ML7, EIPA at indicated concentrations and individual inhibitors were continuously present during following steps of the experiment. (D). Cells where infected with RSV (moi ∼3) or SFV-ZsGreen (moi ∼0.5) for up to 6 hours before FACS analysis of GFP expressing cells. (E). RSV (moi ∼3) was bound to the cells at 4°C followed by 1 h of internalization at 37°C. Cells were trypsinized, fixed and stained with anti-N-AF488 antibody, and the MFI of AF-488 measured by FACS. (F, G). Cells where infected with RSV (moi ∼3) for 6 hours before FACS analysis of GFP expressing cells.
(TIF)

Figure S4 **RSV infection of cells expressing Rab5 and Rab7.** HeLa cells were transiently transfected with a GFP-Rab5 WT, Rab5 Q79L (C/A), Rab5 S34N (D/N), Rab7 WT, Rab7 Q67L (C/A), Rab7 T22N (D/N) expressing constructs. After 12 h of transient expression cells were infected with rrRSV expressing m-RFF for additional 18 h. After fixation cells were imaged with the confocal microscope.
(TIF)

Movie S1 **RSV induces transient blebbing of HeLa cells.** HeLa cells were inoculated with a purified RSV (moi 30) and immediately imaged with Olympus CellR microscope with DIC settings with the 20× objective, 1 frame per 10 sec speed at 37C.
(AVI)

Table S1 **SRM assays used to study F0 (UniProt accession number P03420, FUS_HRSVA).**
(DOCX)

## References

[1] Shadman KA, Wald ER (2011) A review of palivizumab and emerging therapies for respiratory syncytial virus. Expert opinion on biological therapy 11: 1455–1467. 21831008 10.1517/14712598.2011.608062

[2] Bachi T, Howe C (1973) Morphogenesis and ultrastructure of respiratory syncytial virus. Journal of virology 12: 1173–1180. 4128827 10.1128/jvi.12.5.1173-1180.1973 PMC356750

[3] Collins PL, Graham BS (2008) Viral and host factors in human respiratory syncytial virus pathogenesis. Journal of virology 82: 2040–2055. 17928346 10.1128/JVI.01625-07 PMC2258918

[4] Hallak LK, Collins PL, Knudson W, Peeples ME (2000) Iduronic acid-containing glycosaminoglycans on target cells are required for efficient respiratory syncytial virus infection. Virology 271: 264–275. 10860881 10.1006/viro.2000.0293

[5] Techaarpornkul S, Barretto N, Peeples ME (2001) Functional analysis of recombinant respiratory syncytial virus deletion mutants lacking the small hydrophobic and/or attachment glycoprotein gene. Journal of virology 75: 6825–6834. 11435561 10.1128/JVI.75.15.6825-6834.2001 PMC114409

[6] Villenave R, Thavagnanam S, Sarlang S, Parker J, Douglas I, et al (2012) *In vitro* modeling of respiratory syncytial virus infection of pediatric bronchial epithelium, the primary target of infection *in vivo* . Proceedings of the National Academy of Sciences of the United States of America 109: 5040–5045. 22411804 10.1073/pnas.1110203109 PMC3323997

[7] Srinivasakumar N, Ogra PL, Flanagan TD (1991) Characteristics of fusion of respiratory syncytial virus with HEp-2 cells as measured by R18 fluorescence dequenching assay. Journal of virology 65: 4063–4069. 1906550 10.1128/jvi.65.8.4063-4069.1991 PMC248838

[8] Kahn JS, Schnell MJ, Buonocore L, Rose JK (1999) Recombinant vesicular stomatitis virus expressing respiratory syncytial virus (RSV) glycoproteins: RSV fusion protein can mediate infection and cell fusion. Virology 254: 81–91. 9927576 10.1006/viro.1998.9535

[9] Kolokoltsov AA, Deniger D, Fleming EH, Roberts NJ Jr, Karpilow JM, et al (2007) Small interfering RNA profiling reveals key role of clathrin-mediated endocytosis and early endosome formation for infection by respiratory syncytial virus. Journal of virology 81: 7786–7800. 17494077 10.1128/JVI.02780-06 PMC1933373

[10] San-Juan-Vergara H, Sampayo-Escobar V, Reyes N, Cha B, Pacheco-Lugo L, et al (2012) Cholesterol-rich microdomains as docking platforms for respiratory syncytial virus in normal human bronchial epithelial cells. Journal of virology 86: 1832–1843. 22090136 10.1128/JVI.06274-11 PMC3264380

[11] Kwilas AR, Yednak MA, Zhang L, Liesman R, Collins PL, et al (2010) Respiratory syncytial virus engineered to express the cystic fibrosis transmembrane conductance regulator corrects the bioelectric phenotype of human cystic fibrosis airway epithelium *in vitro* . Journal of virology 84: 7770–7781. 20504917 10.1128/JVI.00346-10 PMC2897634

[12] Radhakrishnan A, Yeo D, Brown G, Myaing MZ, Iyer LR, et al (2010) Protein analysis of purified respiratory syncytial virus particles reveals an important role for heat shock protein 90 in virus particle assembly. Molecular & cellular proteomics : MCP 9: 1829–1848. 20530633 10.1074/mcp.M110.001651 PMC2938102

[13] Busetto S, Trevisan E, Patriarca P, Menegazzi R (2004) A single-step, sensitive flow cytofluorometric assay for the simultaneous assessment of membrane-bound and ingested Candida albicans in phagocytosing neutrophils. Cytometry Part A : the journal of the International Society for Analytical Cytology 58: 201–206. 15057974 10.1002/cyto.a.20014

[14] Sakai T, Ohuchi M, Imai M, Mizuno T, Kawasaki K, et al (2006) Dual wavelength imaging allows analysis of membrane fusion of influenza virus inside cells. Journal of virology 80: 2013–2018. 16439557 10.1128/JVI.80.4.2013-2018.2006 PMC1367152

[15] Ivanov AI (2008) Pharmacological inhibition of endocytic pathways: is it specific enough to be useful? Methods in molecular biology 440: 15–33. 18369934 10.1007/978-1-59745-178-9_2

[16] von Kleist L, Stahlschmidt W, Bulut H, Gromova K, Puchkov D, et al (2011) Role of the clathrin terminal domain in regulating coated pit dynamics revealed by small molecule inhibition. Cell 146: 471–484. 21816279 10.1016/j.cell.2011.06.025

[17] Chircop M, Perera S, Mariana A, Lau H, Ma MP, et al (2011) Inhibition of dynamin by dynole 34-2 induces cell death following cytokinesis failure in cancer cells. Molecular cancer therapeutics 10: 1553–1562. 21750222 10.1158/1535-7163.MCT-11-0067

[18] Marsh M, Bolzau E, Helenius A (1983) Penetration of Semliki Forest virus from acidic prelysosomal vacuoles. Cell 32: 931–940. 6831562 10.1016/0092-8674(83)90078-8

[19] Helenius A, Marsh M, White J (1982) Inhibition of Semliki forest virus penetration by lysosomotropic weak bases. The Journal of general virology 58 Pt 1: 47–61. 7142969 10.1099/0022-1317-58-1-47

[20] Mercer J, Helenius A (2009) Virus entry by macropinocytosis. Nature cell biology 11: 510–520. 19404330 10.1038/ncb0509-510

[21] Mercer J, Helenius A (2012) Gulping rather than sipping: macropinocytosis as a way of virus entry. Current opinion in microbiology 15: 490–9. 22749376 10.1016/j.mib.2012.05.016

[22] Norbury CC (2006) Drinking a lot is good for dendritic cells. Immunology 117: 443–451. 16556257 10.1111/j.1365-2567.2006.02335.x PMC1782244

[23] Koivusalo M, Welch C, Hayashi H, Scott CC, Kim M, et al (2010) Amiloride inhibits macropinocytosis by lowering submembranous pH and preventing Rac1 and Cdc42 signaling. The Journal of cell biology 188: 547–563. 20156964 10.1083/jcb.200908086 PMC2828922

[24] Swanson JA (2008) Shaping cups into phagosomes and macropinosomes. Nature reviews Molecular cell biology 9: 639–649. 18612320 10.1038/nrm2447 PMC2851551

[25] Jiang J, Kolpak AL, Bao ZZ (2010) Myosin IIB isoform plays an essential role in the formation of two distinct types of macropinosomes. Cytoskeleton 67: 32–42. 19743471 10.1002/cm.20419 PMC2825287

[26] Rupper A, Lee K, Knecht D, Cardelli J (2001) Sequential activities of phosphoinositide 3-kinase, PKB/Aakt, and Rab7 during macropinosome formation in Dictyostelium. Molecular biology of the cell 12: 2813–2824. 11553719 10.1091/mbc.12.9.2813 PMC59715

[27] Stenmark H, Parton RG, Steele-Mortimer O, Lutcke A, Gruenberg J, et al (1994) Inhibition of rab5 GTPase activity stimulates membrane fusion in endocytosis. The EMBO journal 13: 1287–1296. 8137813 10.1002/j.1460-2075.1994.tb06381.x PMC394944

[28] Kerr MC, Lindsay MR, Luetterforst R, Hamilton N, Simpson F, et al (2006) Visualisation of macropinosome maturation by the recruitment of sorting nexins. Journal of cell science 119: 3967–3980. 16968745 10.1242/jcs.03167

[29] Gonzalez-Reyes L, Ruiz-Arguello MB, Garcia-Barreno B, Calder L, Lopez JA, et al (2001) Cleavage of the human respiratory syncytial virus fusion protein at two distinct sites is required for activation of membrane fusion. Proceedings of the National Academy of Sciences of the United States of America 98: 9859–9864. 11493675 10.1073/pnas.151098198 PMC55543

[30] Zimmer G, Budz L, Herrler G (2001) Proteolytic activation of respiratory syncytial virus fusion protein. Cleavage at two furin consensus sequences. The Journal of biological chemistry 276: 31642–31650. 11418598 10.1074/jbc.M102633200

[31] Lutschg V, Boucke K, Hemmi S, Greber UF (2011) Chemotactic antiviral cytokines promote infectious apical entry of human adenovirus into polarized epithelial cells. Nature communications 2: 391. 10.1038/ncomms1391 PMC7091692 21750545

[32] Lamb RA, Paterson RG, Jardetzky TS (2006) Paramyxovirus membrane fusion: lessons from the F and HN atomic structures. Virology 344: 30–37. 16364733 10.1016/j.virol.2005.09.007 PMC7172328

[33] Chang A, Dutch RE (2012) Paramyxovirus fusion and entry: multiple paths to a common end. Viruses 4: 613–636. 22590688 10.3390/v4040613 PMC3347325

[34] Rasmusson BJ, Flanagan TD, Turco SJ, Epand RM, Petersen NO (1998) Fusion of Sendai virus and individual host cells and inhibition of fusion by lipophosphoglycan measured with image correlation spectroscopy. Biochimica et biophysica acta 1404: 338–352. 9739163 10.1016/s0167-4889(98)00082-2

[35] Diederich S, Thiel L, Maisner A (2008) Role of endocytosis and cathepsin-mediated activation in Nipah virus entry. Virology 375: 391–400. 18342904 10.1016/j.virol.2008.02.019 PMC7103400

[36] Cantin C, Holguera J, Ferreira L, Villar E, Munoz-Barroso I (2007) Newcastle disease virus may enter cells by caveolae-mediated endocytosis. The Journal of general virology 88: 559–569. 17251575 10.1099/vir.0.82150-0

[37] Frecha C, Levy C, Costa C, Negre D, Amirache F, et al (2011) Measles virus glycoprotein-pseudotyped lentiviral vector-mediated gene transfer into quiescent lymphocytes requires binding to both SLAM and CD46 entry receptors. Journal of virology 85: 5975–5985. 21450813 10.1128/JVI.00324-11 PMC3126293

[38] Pernet O, Pohl C, Ainouze M, Kweder H, Buckland R (2009) Nipah virus entry can occur by macropinocytosis. Virology 395: 298–311. 19854459 10.1016/j.virol.2009.09.016

[39] West MA, Bretscher MS, Watts C (1989) Distinct endocytotic pathways in epidermal growth factor-stimulated human carcinoma A431 cells. The Journal of cell biology 109: 2731–2739. 2556406 10.1083/jcb.109.6.2731 PMC2115909

[40] Thomas KW, Monick MM, Staber JM, Yarovinsky T, Carter AB, et al (2002) Respiratory syncytial virus inhibits apoptosis and induces NF-kappa B activity through a phosphatidylinositol 3-kinase-dependent pathway. The Journal of biological chemistry 277: 492–501. 11687577 10.1074/jbc.M108107200

[41] San-Juan-Vergara H, Peeples ME, Lockey RF, Mohapatra SS (2004) Protein kinase C-alpha activity is required for respiratory syncytial virus fusion to human bronchial epithelial cells. Journal of virology 78: 13717–13726. 15564481 10.1128/JVI.78.24.13717-13726.2004 PMC533893

[42] Racoosin EL, Swanson JA (1993) Macropinosome maturation and fusion with tubular lysosomes in macrophages. The Journal of cell biology 121: 1011–1020. 8099075 10.1083/jcb.121.5.1011 PMC2119679

[43] Kielian M, Rey FA (2006) Virus membrane-fusion proteins: more than one way to make a hairpin. Nature reviews Microbiology 4: 67–76. 16357862 10.1038/nrmicro1326 PMC7097298

[44] Chandran K, Sullivan NJ, Felbor U, Whelan SP, Cunningham JM (2005) Endosomal proteolysis of the Ebola virus glycoprotein is necessary for infection. Science 308: 1643–1645. 15831716 10.1126/science.1110656 PMC4797943

[45] Simmons G, Reeves JD, Rennekamp AJ, Amberg SM, Piefer AJ, et al (2004) Characterization of severe acute respiratory syndrome-associated coronavirus (SARS-CoV) spike glycoprotein-mediated viral entry. Proceedings of the National Academy of Sciences of the United States of America 101: 4240–4245. 15010527 10.1073/pnas.0306446101 PMC384725

[46] Diederich S, Sauerhering L, Weis M, Altmeppen H, Schaschke N, et al (2012) Activation of the Nipah virus fusion protein in MDCK cells is mediated by cathepsin B within the endosome-recycling compartment. Journal of virology 86: 3736–3745. 22278224 10.1128/JVI.06628-11 PMC3302499

[47] Pager CT, Craft WW Jr, Patch J, Dutch RE (2006) A mature and fusogenic form of the Nipah virus fusion protein requires proteolytic processing by cathepsin L. Virology 346: 251–257. 16460775 10.1016/j.virol.2006.01.007 PMC7111743

[48] Gruenert DC, Basbaum CB, Widdicombe JH (1990) Long-term culture of normal and cystic fibrosis epithelial cells grown under serum-free conditions. *In vitro* cellular & developmental biology : journal of the Tissue Culture Association 26: 411–418. 1693142 10.1007/BF02623833

[49] Spuul P, Balistreri G, Kaariainen L, Ahola T (2010) Phosphatidylinositol 3-kinase-, actin-, and microtubule-dependent transport of Semliki Forest Virus replication complexes from the plasma membrane to modified lysosomes. Journal of virology 84: 7543–7557. 20484502 10.1128/JVI.00477-10 PMC2897599

[50] Guerrero-Plata A, Casola A, Suarez G, Yu X, Spetch L, et al (2006) Differential response of dendritic cells to human metapneumovirus and respiratory syncytial virus. American journal of respiratory cell and molecular biology 34: 320–329. 16284360 10.1165/rcmb.2005-0287OC PMC2644197

[51] Ueba O (1978) Respiratory syncytial virus. I. Concentration and purification of the infectious virus. Acta medica Okayama 32: 265–272. 153087

[52] Engel S, Heger T, Mancini R, Herzog F, Kartenbeck J, et al (2011) Role of endosomes in simian virus 40 entry and infection. Journal of virology 85: 4198–4211. 21345959 10.1128/JVI.02179-10 PMC3126231

