# Supplementary figures and images for: Host Cell Entry of Respiratory Syncytial Virus Involves Macropinocytosis Followed by Proteolytic Activation of the F Protein
Source: PLoS Pathog. 2013 Apr 11;9(4):e1003309. doi: 10.1371/journal.ppat.1003309 (PMC3623752; doi:10.1371/journal.ppat.1003309)

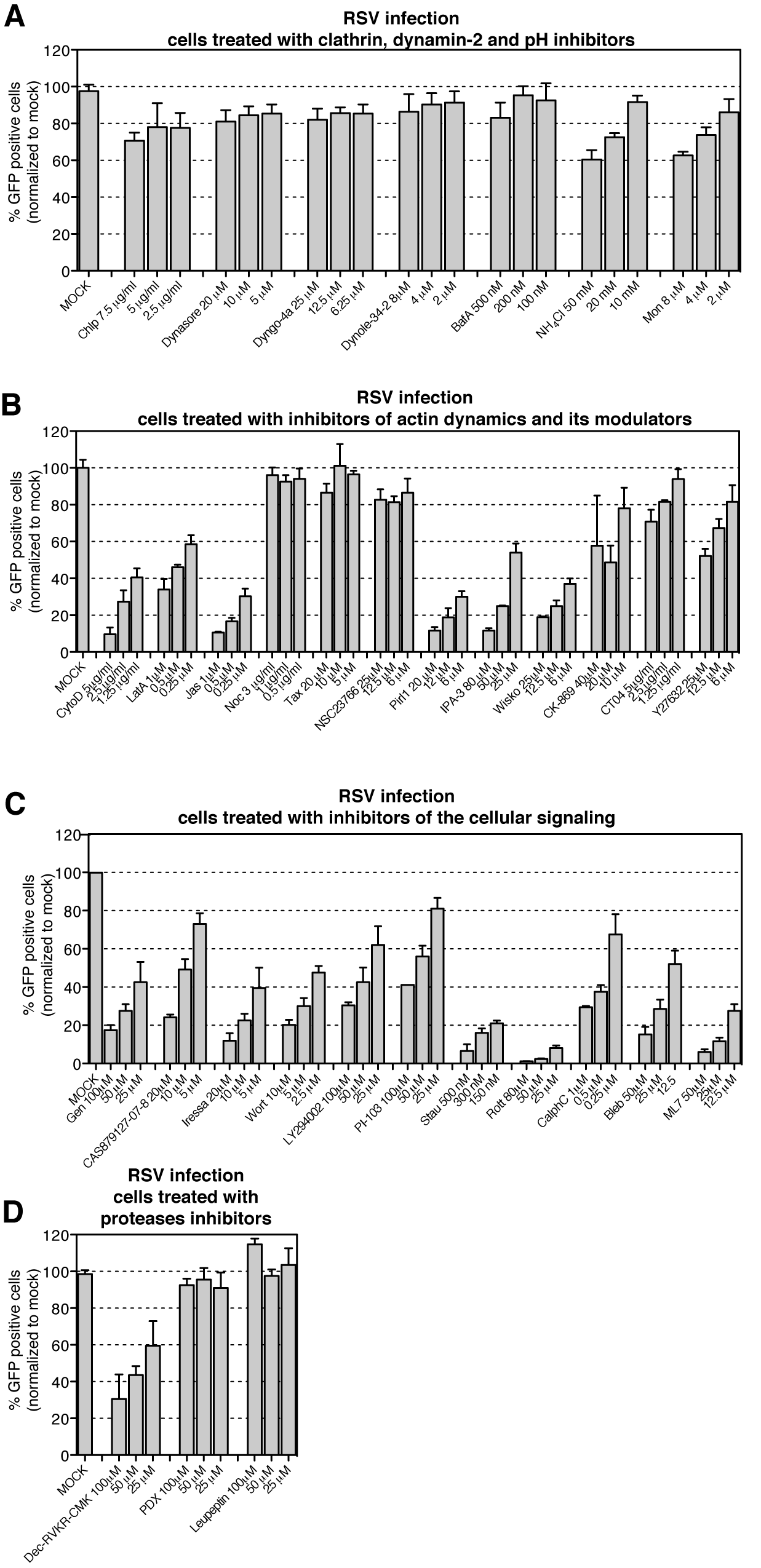

Supplement: Figure S1 — Dose dependent influence of inhibitors on the RSV infection. HeLa cells were pretreated with solvent (MOCK), or (A) chlorpromazine (Chlp), dynasore, dyngo-4a, dynol-34-2, bafilomycin A (BafA), ammonium chloride (NH4Cl), monensin (Mon) (B) cytochalasin D (CytoD), latrunculin A (LatA), jasplakinolide (Jas), nocodazole (Noc), taxol (Tax), NCS23766, pirl1, IPA-3, wiskostatin (Wisko), CK-869, CT04, Y24632 (C) genistein (Gen), CAS879127-07-8, iressa, wortmannin (Wort), LY294002, PI-103, staurosporine (Stau), rottlerin (Rott), calphostin C (CalphC), blebbistatin (Bleb), ML7, (D) dec-RVKR-CMK, PDX or Leupeptin at indicated concentrations and individual inhibitor were continuously present at each step of the experiment. (A–D). Cells were infected with RSV (moi ∼3) for 6 hours, before FACS analysis of GFP expressing cells. (TIF) [file ppat.1003309.s001.tif]

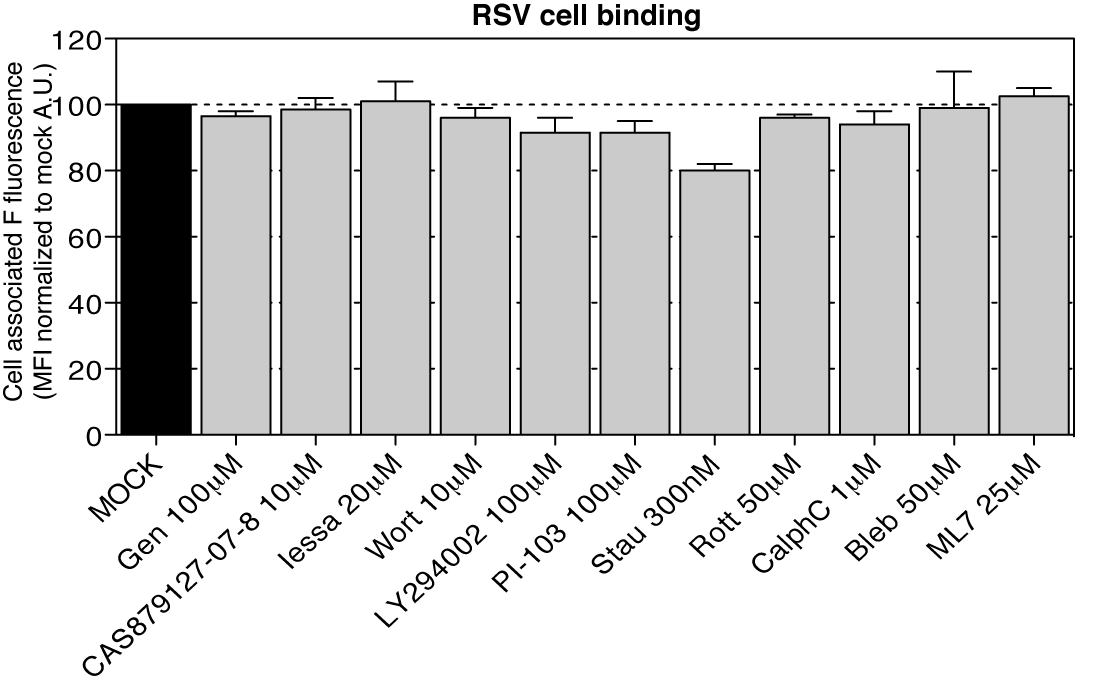

Supplement: Figure S2 — RSV cell binding to cells treated with inhibitors. HeLa cells were pretreated with solvent (MOCK), genistein (Gen), CAS879127-07- 8, iressa, wortmannin (Wort), LY294002, PI-103, staurosporine (Stau), rottlerin (Rott), calphostin C (CalphC), blebbistatin (Bleb), ML7 at indicated concentrations and individual inhibitor were continuously present at each step of the experiment. Cells were chilled on ice and RSV (moi ∼3) was bound to the cells in cold for 1 h. Cells were fixed, permeabilized, stain with anti-F-AF488 antibody, and the MFI of AF-488 measured by FACS. (TIF) [file ppat.1003309.s002.tif]

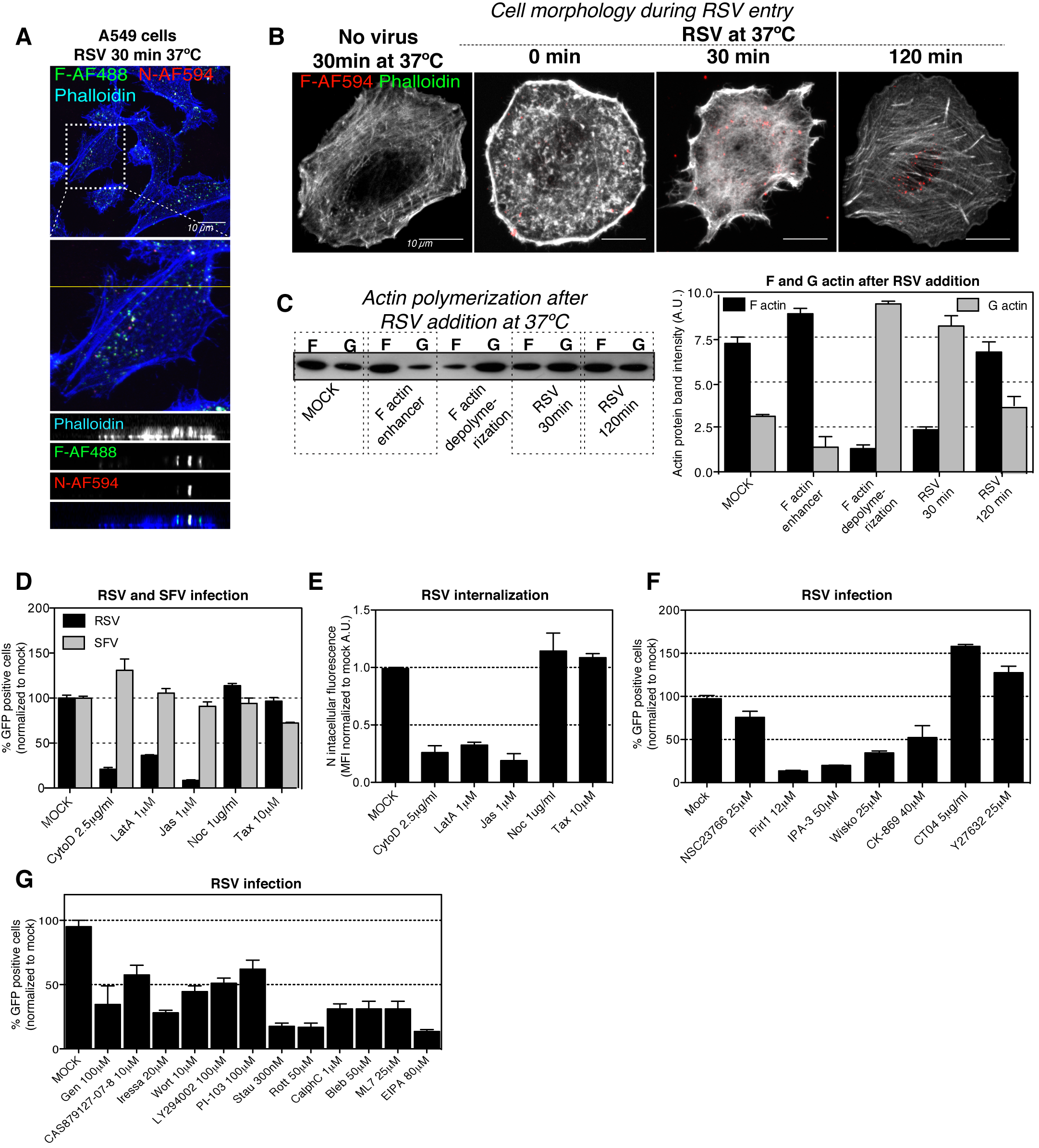

Supplement: Figure S3 — RSV enters A549 cells by macropinocytosis. (A). RSV (moi ∼0.5) was bound to A549 cells at 4°C followed by 30 min at 37°C. Cells were by IIF with anti-F-AF488 (green), anti-N-AF594 (red), and phalloidin- AF647 (pseudocolored white) for confocal microscopy as, and Z-stack image series acquired. The orthogonal views of image Z-stacks (pseudo-colored white) were generated with ImageJ. (B). RSV (moi ∼0.5) was bound to A549 cells at 4°C, virus inoculum was washed, cells warmed to 37°C, fixed at indicated times, and stained with phalloidin-AF488 (pseudo colored white) and anti-F-AF647 (red) antibody. Images represent Z-stack projections acquired with a confocal microscope. (C). RSV (moi ∼30) was incubated with A549 cells for 30 or 120 min at 37°C. Samples were processed according to the kit manufacturer's protocol (Cytoskeleton Inc.). Controls included mock-treated cells and cells either treated with F actin enhancer or F actin depolymerizing agent. (left) The F and G actin fractions were resolved by SDS-PAGE and western blots probed with anti-actin antibody. (right) Quantification of actin protein bands intensities by densitometry. A549 cells were pretreated with solvent (MOCK) or (D–E) cytochalasin D (CytoD), latrunculin A (LatA), jasplakinolide (Jas), nocodazole (Noc), taxol (Tax), (F) NCS23766, pirl1, IPA-3, wiskostatin (Wisko), CK-869, CT04, Y24632, (G) genistein (Gen), CAS879127-07-8, Iressa, wortmannin (Wort), LY294002, PI-103, staurosporine (Stau), rottlerin (Rott), calphostin C (CalphC), blebbistatin (Bleb), ML7, EIPA at indicated concentrations and individual inhibitors were continuously present during following steps of the experiment. (D). Cells where infected with RSV (moi ∼3) or SFV-ZsGreen (moi ∼0.5) for up to 6 hours before FACS analysis of GFP expressing cells. (E). RSV (moi ∼3) was bound to the cells at 4°C followed by 1 h of internalization at 37°C. Cells were trypsinized, fixed and stained with anti-N-AF488 antibody, and the MFI of AF-488 measured by FA [file ppat.1003309.s003.tif]

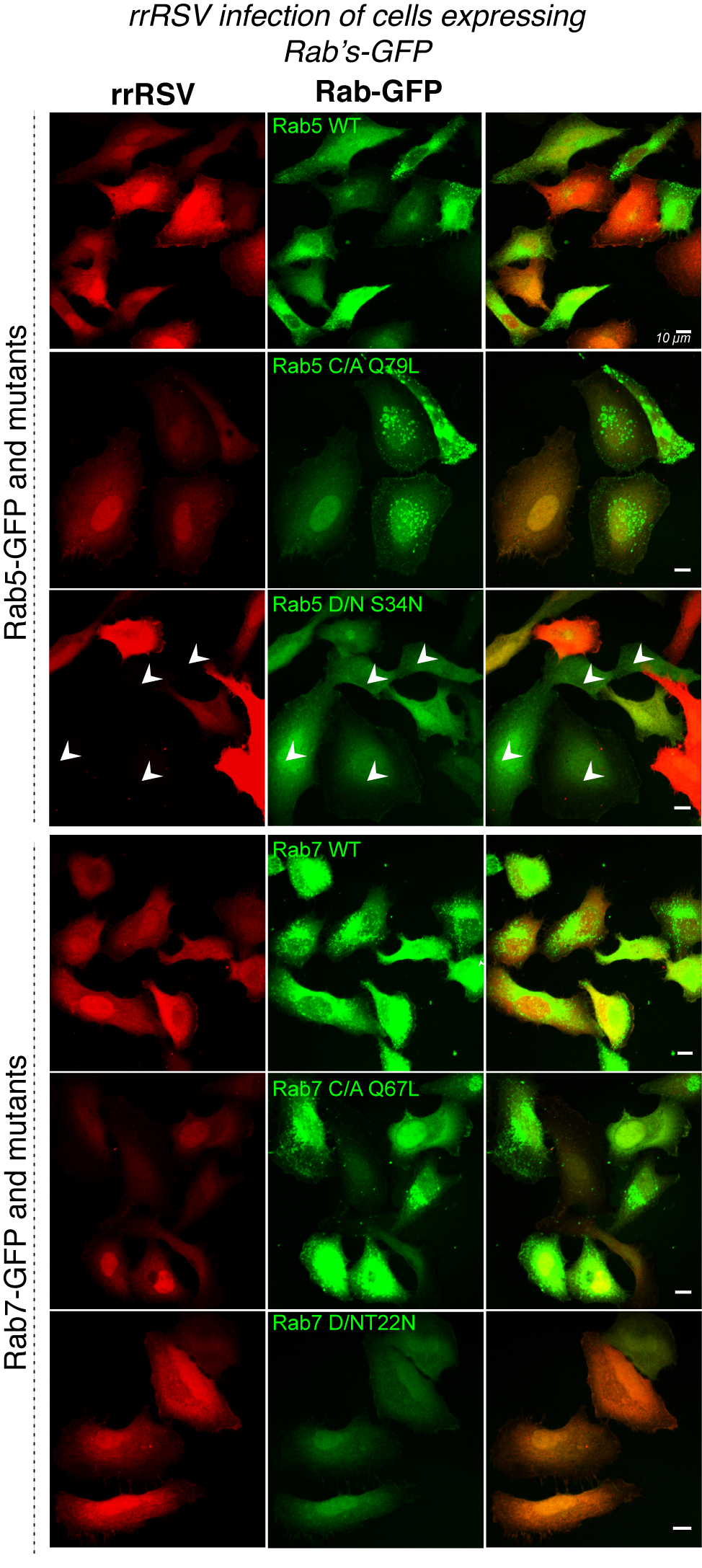

Supplement: Figure S4 — RSV infection of cells expressing Rab5 and Rab7. HeLa cells were transiently transfected with a GFP-Rab5 WT, Rab5 Q79L (C/A), Rab5 S34N (D/N), Rab7 WT, Rab7 Q67L (C/A), Rab7 T22N (D/N) expressing constructs. After 12 h of transient expression cells were infected with rrRSV expressing m-RFF for additional 18 h. After fixation cells were imaged with the confocal microscope. (TIF) [file ppat.1003309.s004.tif]
