# Supplementary material for: Host Cell Entry of Respiratory Syncytial Virus Involves Macropinocytosis Followed by Proteolytic Activation of the F Protein
Source: PLoS Pathog. 2013 Apr 11;9(4):e1003309. doi: 10.1371/journal.ppat.1003309 (PMC3623752; doi:10.1371/journal.ppat.1003309)
Supplement: Table S1 — SRM assays used to study F0 (UniProt accession number P03420, FUS_HRSVA). (DOCX) [file ppat.1003309.s006.docx]

| Supporting Table S1: SRM assays used to study F0 (UniProt accession number P03420, FUS_HRSVA) |
| --- |

| **Q1^1^** | **Q3^2^** | **Precursor sequence** | **Precursor Charge** | **Fragment Charge** | **Ion Type** | **Label** | **CE^3^** |
| --- | --- | --- | --- | --- | --- | --- | --- |
| 570.959251 | 1215.58267 | ELPRFMNYTLNNAK | 3 | 3 | y10 | light | 28.7 |
| 570.959251 | 1068.514256 | ELPRFMNYTLNNAK | 3 | 3 | y9 | light | 28.7 |
| 570.959251 | 937.473771 | ELPRFMNYTLNNAK | 3 | 3 | y8 | light | 28.7 |
| 570.959251 | 823.430844 | ELPRFMNYTLNNAK | 3 | 3 | y7 | light | 28.7 |
| 570.959251 | 660.367515 | ELPRFMNYTLNNAK | 3 | 3 | y6 | light | 28.7 |
| 570.959251 | 791.413942 | ELPRFMNYTLNNAK | 3 | 2 | y13 | light | 28.7 |
| 570.959251 | 734.87191 | ELPRFMNYTLNNAK | 3 | 2 | y12 | light | 28.7 |
| 570.959251 | 686.345528 | ELPRFMNYTLNNAK | 3 | 2 | y11 | light | 28.7 |
| 570.959251 | 608.294973 | ELPRFMNYTLNNAK | 3 | 2 | y10 | light | 28.7 |
| 570.959251 | 490.250366 | ELPRFMNYTLNNAK | 3 | 2 | y12 | light | 28.7 |
| 570.959251 | 643.356222 | ELPRFMNYTLNNAK | 3 | 3 | b5 | light | 28.7 |
| 570.959251 | 774.396707 | ELPRFMNYTLNNAK | 3 | 3 | b6 | light | 28.7 |
| 570.959251 | 888.439634 | ELPRFMNYTLNNAK | 3 | 3 | b7 | light | 28.7 |
| 570.959251 | 1051.502963 | ELPRFMNYTLNNAK | 3 | 3 | b8 | light | 28.7 |
| 570.959251 | 1152.550641 | ELPRFMNYTLNNAK | 3 | 3 | b9 | light | 28.7 |
| 570.959251 | 1265.634705 | ELPRFMNYTLNNAK | 3 | 3 | b10 | light | 28.7 |
| 570.959251 | 576.778959 | ELPRFMNYTLNNAK | 3 | 2 | b9 | light | 28.7 |
| 570.959251 | 633.320991 | ELPRFMNYTLNNAK | 3 | 2 | b10 | light | 28.7 |
| 570.959251 | 690.342454 | ELPRFMNYTLNNAK | 3 | 2 | b11 | light | 28.7 |
| 570.959251 | 747.363918 | ELPRFMNYTLNNAK | 3 | 2 | b12 | light | 28.7 |
| 570.959251 | 782.882475 | ELPRFMNYTLNNAK | 3 | 2 | b13 | light | 28.7 |
| 696.36998 | 1203.705562 | FMNYTLNNAKKTNVTLSK | 3 | 3 | y11 | light | 35.5 |
| 696.36998 | 1089.662635 | FMNYTLNNAKKTNVTLSK | 3 | 3 | y10 | light | 35.5 |
| 696.36998 | 1018.625521 | FMNYTLNNAKKTNVTLSK | 3 | 3 | y9 | light | 35.5 |
| 696.36998 | 890.530558 | FMNYTLNNAKKTNVTLSK | 3 | 3 | y8 | light | 35.5 |
| 696.36998 | 762.435595 | FMNYTLNNAKKTNVTLSK | 3 | 3 | y7 | light | 35.5 |
| 696.36998 | 970.517124 | FMNYTLNNAKKTNVTLSK | 3 | 2 | y17 | light | 35.5 |
| 696.36998 | 904.996882 | FMNYTLNNAKKTNVTLSK | 3 | 2 | y16 | light | 35.5 |
| 696.36998 | 847.975418 | FMNYTLNNAKKTNVTLSK | 3 | 2 | y15 | light | 35.5 |
| 696.36998 | 766.443754 | FMNYTLNNAKKTNVTLSK | 3 | 2 | y14 | light | 35.5 |
| 696.36998 | 715.919915 | FMNYTLNNAKKTNVTLSK | 3 | 2 | y13 | light | 35.5 |
| 696.36998 | 770.354173 | FMNYTLNNAKKTNVTLSK | 3 | 3 | b6 | light | 35.5 |
| 696.36998 | 884.397101 | FMNYTLNNAKKTNVTLSK | 3 | 3 | b7 | light | 35.5 |
| 696.36998 | 998.440028 | FMNYTLNNAKKTNVTLSK | 3 | 3 | b8 | light | 35.5 |
| 696.36998 | 1069.477142 | FMNYTLNNAKKTNVTLSK | 3 | 3 | b9 | light | 35.5 |
| 696.36998 | 1197.572105 | FMNYTLNNAKKTNVTLSK | 3 | 3 | b10 | light | 35.5 |
| 696.36998 | 1325.667068 | FMNYTLNNAKKTNVTLSK | 3 | 3 | b11 | light | 35.5 |
| 696.36998 | 1426.714747 | FMNYTLNNAKKTNVTLSK | 3 | 3 | b12 | light | 35.5 |
| 696.36998 | 713.861011 | FMNYTLNNAKKTNVTLSK | 3 | 2 | b12 | light | 35.5 |
| 696.36998 | 770.882475 | FMNYTLNNAKKTNVTLSK | 3 | 2 | b13 | light | 35.5 |
| 696.36998 | 820.416682 | FMNYTLNNAKKTNVTLSK | 3 | 2 | b14 | light | 35.5 |
| 696.36998 | 870.940521 | FMNYTLNNAKKTNVTLSK | 3 | 2 | b15 | light | 35.5 |
| 696.36998 | 927.482553 | FMNYTLNNAKKTNVTLSK | 3 | 2 | b16 | light | 35.5 |
| 696.36998 | 970.998567 | FMNYTLNNAKKTNVTLSK | 3 | 2 | b17 | light | 35.5 |

| ^1^Q1: Mass-to-charge ratio (m/z) of the peptide ion used as Q1 value. ^2^Q3: Mass-to-charge ratio (m/z) of the fragment ion used as Q3 value. ^3^CE: Applied collision energy |
| --- |
|  |
